# A large new Middle Jurassic ichthyosaur shows the importance of body size evolution in the origin of the Ophthalmosauria

**DOI:** 10.1186/s12862-024-02208-3

**Published:** 2024-03-16

**Authors:** Feiko Miedema, Dylan Bastiaans, Torsten M. Scheyer, Christian Klug, Erin E. Maxwell

**Affiliations:** 1 Staatliches Museum für Naturkunde Stuttgart, Rosenstein 1, Stuttgart, 70191 Germany; 2 Universität Zürich, Paläontologisches Institut, Karl Schmid-Strasse 4, Zürich, 8006 Switzerland; 3 NHMB: Naturhistorisches Museum Basel, Augustinergasse 2, Basel, 4001 Switzerland

**Keywords:** Ichthyosauria, Ophthalmosauria, Bajocian, Body size evolution, Middle Jurassic

## Abstract

The Middle Jurassic is an important time period for the evolutionary history of marine reptiles as it represented a transitional phase for many clades. Notably, in ichthyosaurs, many early parvipelvian taxa went extinct. The Middle Jurassic saw the emergence of the derived Ophthalmosauria, ultimately becoming the dominant ichthyosaurian clade by the end of the epoch. Even though this is an important period in the evolutionary history of Ophthalmosauria, our understanding remains limited in terms of morphology and taxonomy due to the scarcity of vertebrate-bearing strata. Here we present a large new ichthyosaur from the Bajocian of Switzerland, represented by an almost complete skull with 3D-preserved bones, the (inter)clavicles and a large portion of the postcranial skeleton. After CT- and surface scanning, we reconstructed the 3D in vivo morphology. Our morphological observations and phylogenetic analyses show that the new taxon named *Argovisaurus martafernandezi* is nested at the base of the Ophthalmosauria. The holotype and only known specimen of *Argovisaurus* likely represents an adult individual. Bajocian members of the Ophthalmosauria (*Mollesaurus* and *Argovisaurus*) were large-bodied animals, a trait typically associated with the more derived Platypterygiinae. This hints at the importance of a large body size early in ophthalmosaurian evolution.

**LSID**: urn:lsid:zoobank.org:act:C3312628-1544-4B87-BBE3-B12346A30BE3

**LSID**: urn:lsid:zoobank.org:act:23C2BD71-8CF0-4D99-848A-0D631518415B

**Supplementary Information:**

The online version contains supplementary material available at 10.1186/s12862-024-02208-3.

## Introduction

Ichthyosaurs are a major marine reptile clade that dominated marine ecosystems throughout much of the Mesozoic [1, 2]. Well-known for their thunniform body shape and viviparous reproductive strategy [3, 4], they were represented by numerous clades in the Early Jurassic. By the Late Jurassic and Cretaceous, however, the clade featured members almost exclusively belonging to the Ophthalmosauria [4–8]. The transition of these two distinct faunas occurred in the Middle Jurassic. Phylogenetic shifts as observed in ichthyosaurs mirror similar trends in Thalattosuchia and Plesiosauria. Early plesiosaurs, rhomaleosaurids and pelagic teleosaurids were replaced by more derived pliosaurids, cryptoclidid plesiosaurs and metriorhynchids (summarized in [5]). Thus, the Middle Jurassic represents an important time interval with a distinct faunal turnover of marine higher trophic consumers [5, 9]. While many Early- and Late Jurassic localities with well-preserved marine vertebrate fossils are known, especially in Europe (e.g., Jurassic coast, UK; Holzmaden, Solnhofen, Germany), the same cannot be said for the Middle Jurassic (Aalenian-Bathonian interval). Very few localities, and few specimens have so far been recovered from this interval. This is potentially due to the loss of shelf habitats in a regressive event that persisted throughout most of this epoch [9]. The only exception is potentially the Los Molles Formation in Argentina which has yielded articulated material of both ichthyosaurs and plesiosaurs [10–12]. Due to this limited fossil record, our understanding of the evolutionary transition of marine reptile clades from the Early- to the Late Jurassic is limited. The Middle Jurassic ichthyosaur sample consists of some material from the Aalenian of Germany, including two complete specimens, and some incomplete material of Bajocian age from Argentina, Alaska and western Europe [5, 13–15]. Only three taxa have so far been described from this interval: *Mollesaurus periallus*, *Chacaicosaurus* (= *Stenopterygius*?) *cayi* and *Stenopterygius aaleniensis* [11–13]. The Callovian (latest Middle Jurassic) presents a slight improvement in the European record, with the Oxford Clay Lagerstätte of the UK yielding abundant ichthyosaurian material that is almost exclusively referred to *Ophthalmosaurus icenicus* [16]. Globally, however, the record remains fragmentary. This Middle Jurassic sampling bias has limited our understanding of a key moment in ichthyosaur evolutionary history: the origination and early radiation of the Ophthalmosauria. Switzerland has already yielded bountiful ichthyosaur material. It is probably best-known for its Middle Triassic specimens, heralding from the UNESCO World Heritage site of Monte San Giorgio [17–21], although material from other time periods is also known [22–25]. Here, we report on a large new genus of ichthyosaur of Bajocian age from Canton Aargau, Switzerland. The new taxon likely represents an early member of the derived Ophthalmosauria and thereby yields important information to close the evolutionary gap between Early- and Late Jurassic ichthyosaurs as well as information regarding ecosystem development in the early Middle Jurassic.

### Material

**Fig. 1:**
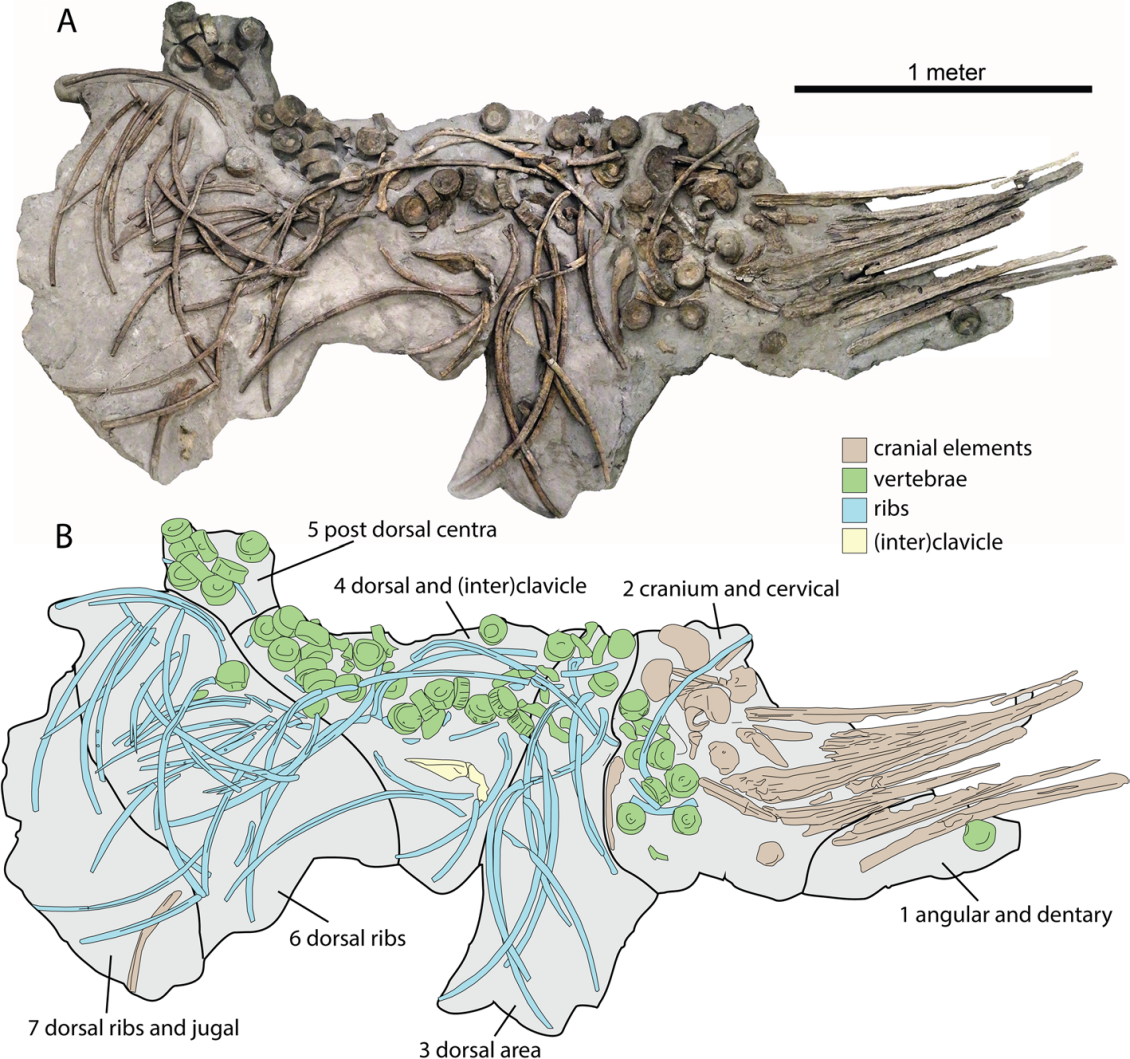
Overview of the (partially interpreted) taphonomic situation and exhibition setting of *Argovisaurus martafernandezi*, holotype PIMUZ A/III 5279. **A** overview of all the restored blocks restored as exhibited. **B** interpretative drawing of the overview showing the separately collected blocks. Notes: The exact location of blocks 1 and 5 is unknown, they have been restored to their most logical taphonomic location. The right angular and left jugal have been separately collected and placed as best guesses

PIMUZ A/III 5279 was discovered over twenty years ago in the old quarry of Oberegg north of Auenstein in Canton Aargau (Switzerland). The skeleton was discovered roughly in the center of the northern slope of the quarry, a few meters west of a north-south running fault (coordinates N47.42438554613916, E8.142617724821447). Most of it was originally excavated by private collector Elmar Meier and acquired by the PIMUZ in 2019. Subsequent expeditions by the PIMUZ to the original location unearthed additional material, namely the right angular and right jugal. The main part of the specimen was extracted in multiple blocks and many small fragments (Fig. 1). PIMUZ A/III 5279 consists of numerous disarticulated vertebral centra, neural arches and ribs, two partial clavicles, and a nearly complete disarticulated cranium (Figs. 1 and 6). Preserved cranial elements include an almost complete braincase, the parietals, some of the circumorbital area, a large portion of the palate, and most of the jaws including complete premaxillae and dentaries. Most of the vertebrae and ribs are from the cervical and dorsal regions but there are a few anterior caudal elements present. The skull and selected postcranial bones such as cervical vertebrae and the clavicles are completely prepared free of matrix. Much of the posterior skeleton remains partially embedded in the matrix. External bone tissue is pristinely preserved, but in many elements, extensive remineralization of endosteal tissue has occurred, which hampered CT-scanning and may hamper potential future histological studies.

### Geological setting

**Fig. 2:**
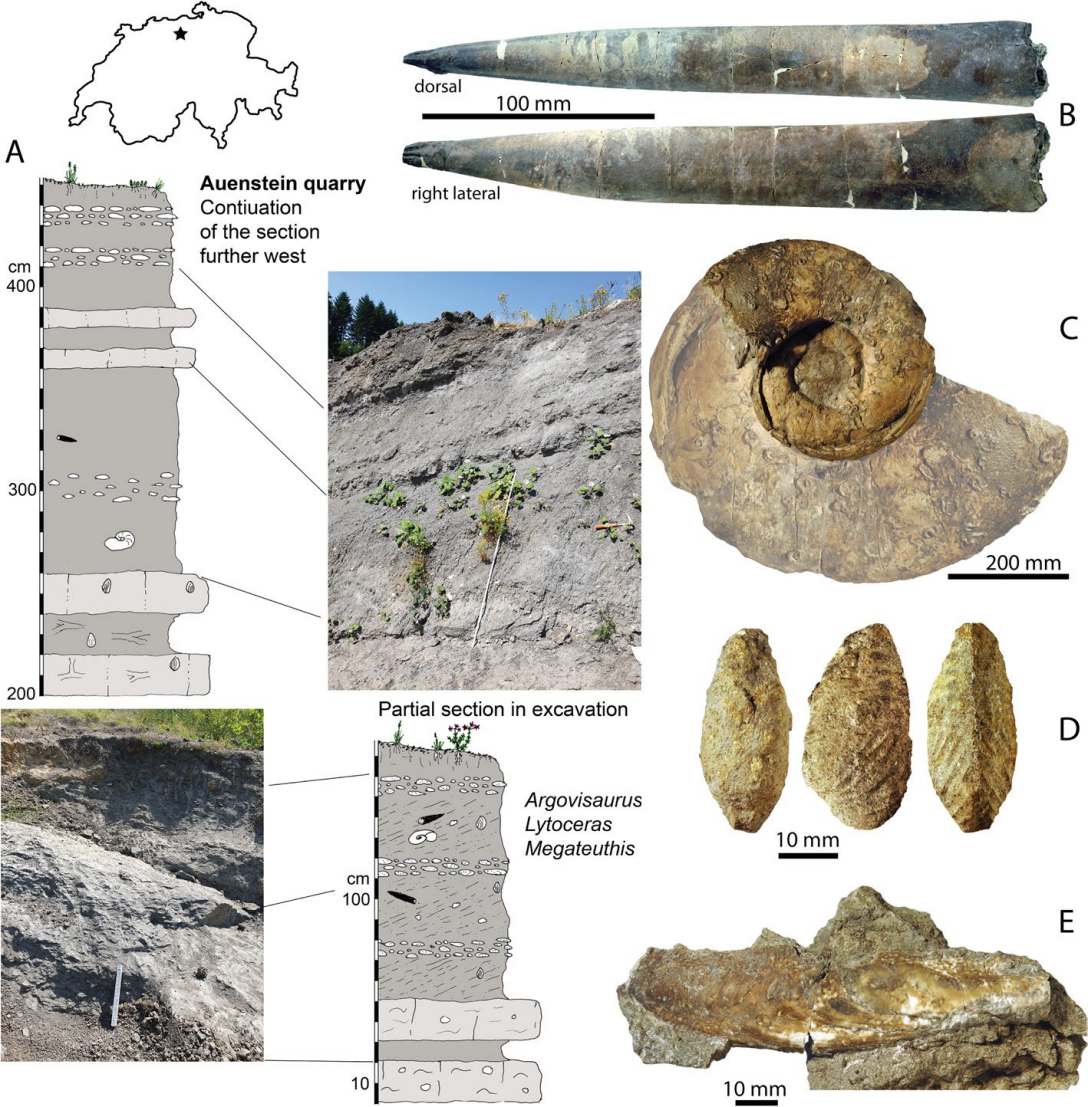
The Auenstein quarry locality. **A** Location, stratigraphic column and lithology of the Auenstein quarry at Oberegg. **B** Rostrum of *Megateuthis suevica* found in close vicinity to the *Argovisaurus* holotype. **C** Large phragmocone of *Lytoceras* cf. *eudesianum* found in close vicinity to the *Argovisaurus* holotype (Photo was duplicated, magnified and turned to represent the approximate shape and size of the body chamber, of which only the umbilical seam is preserved). Examples of bivalves from the Lower Acuminata beds: **D ***Goniomya literata* (seen in L-R dorsal, left lateral and ventral view) **E ***Gervillella aviculoides*

The studied interval belongs to the lower Hauptrogenstein Formation [26, 27]. It is of middle Bajocian to early Bathonian age. At Auenstein Oberegg, the exposed succession lithostratigraphically comprises (1) marly limestones with ooids, the Lower Acuminata series, (2) massive oolitic limestones of the Lower Hauptrogenstein (Untere oolitische Serien sensu [26]) representing an ebb-tidal delta prograding southward, (3) marly calcarenitic-tempestitic wackestones of the Parkinsoni-Schichten (approximately coeval to the ‘Obere Acuminata-Schichten' in western Switzerland) with limestone nodules, abundant bivalves (often in life-position), trace fossils and various cephalopods, (4) overlain by a cross-bedded ‘sand wave’-complex of the so-called ‘Spatkalk’, recording flow towards the south, capped by (5) a condensation level (‘Schellenbrücke-Schicht’, lower Oxfordian) at the Middle to Upper Jurassic boundary [28], and (6) above that, the sponge-rich Birmensdorf and Effingen members of the Wildegg Formation (Fig. 2).

PIMUZ A/III 5279 was discovered in the marly facies of the Lower Acuminata beds [27, 29], which are about 8 m thick (Fig. 2A). At the Oberegg site, the facies is clayey to marly, with layers having a slightly elevated carbonate content, horizons with limestone concretions, usually containing some ooids and some calcarenitic beds (Fig. 2A). The marls are rich in sand and silt, indistinctly bedded and bioturbated. Sedimentary structures of some calcarenitic strata imply their tempestitic origin [29]. These sedimentological features suggest a depositional water depth above the storm wave base on a carbonate ramp.

PIMUZ A/III 5279 was found in association with ubiquitous echinoderm ossicles, abundant bivalves, some belemnites (*Megateuthis suevica*), ichnofossils (e.g., *Thalassinoides*, *Teichichnus*?, *Lockeia*?), some brachiopods (*Terebratula furcilensis, T. movelierensis*), and rare nautilids, ammonites and echinoids. Bivalves are quite abundant in the same layer although not always nicely preserved (Fig. 2D-E). Specimens of the infaunal genera *Homomya*, *Pleuromya*, and *Goniomya* could be identified. These taxa confirm a reasonably soft paleosubstrate. Concerning the epifauna, *Praeexogyra acuminata*, bakevelliids, *Gervillella,* and trigoniids were recorded.

Conchs of ectocochleate cephalopods are not very common, in the host stratum but several nautilid conchs were found; these were usually deformed during sediment compaction because they were obliquely to vertically embedded. Only one large ammonite, which was provisionally identified as *Lytoceras* cf. *eudesianum* was recovered (Fig. 2C).

Unfortunately, none of these finds are precise age indicators. The presence of *Megateuthis* strongly suggests a Bajocian age, but not more specific than that. Lower Acuminata beds further northwest contain ammonites that indicate the subfurcatum/niortense zone of the Middle Bajocian [30]. Palynological data of the lower part of the marly interval (Obere Acuminata-Schichten) confirm a parkinsoni zone age, whereas for the upper part (Parkinsoni-Schichten), an early Bathonian age was encountered by both palynological data and finds of index fossils such as *Procerites sp.* (basal zigzag zone) and *Parkinsonia fretensis* (upper zigzag zone) [26]. We therefore consider a Middle to Late Bajocian age the most parsimonious.

## Methods

### Preparation and CT-scanning

**Fig. 3:**
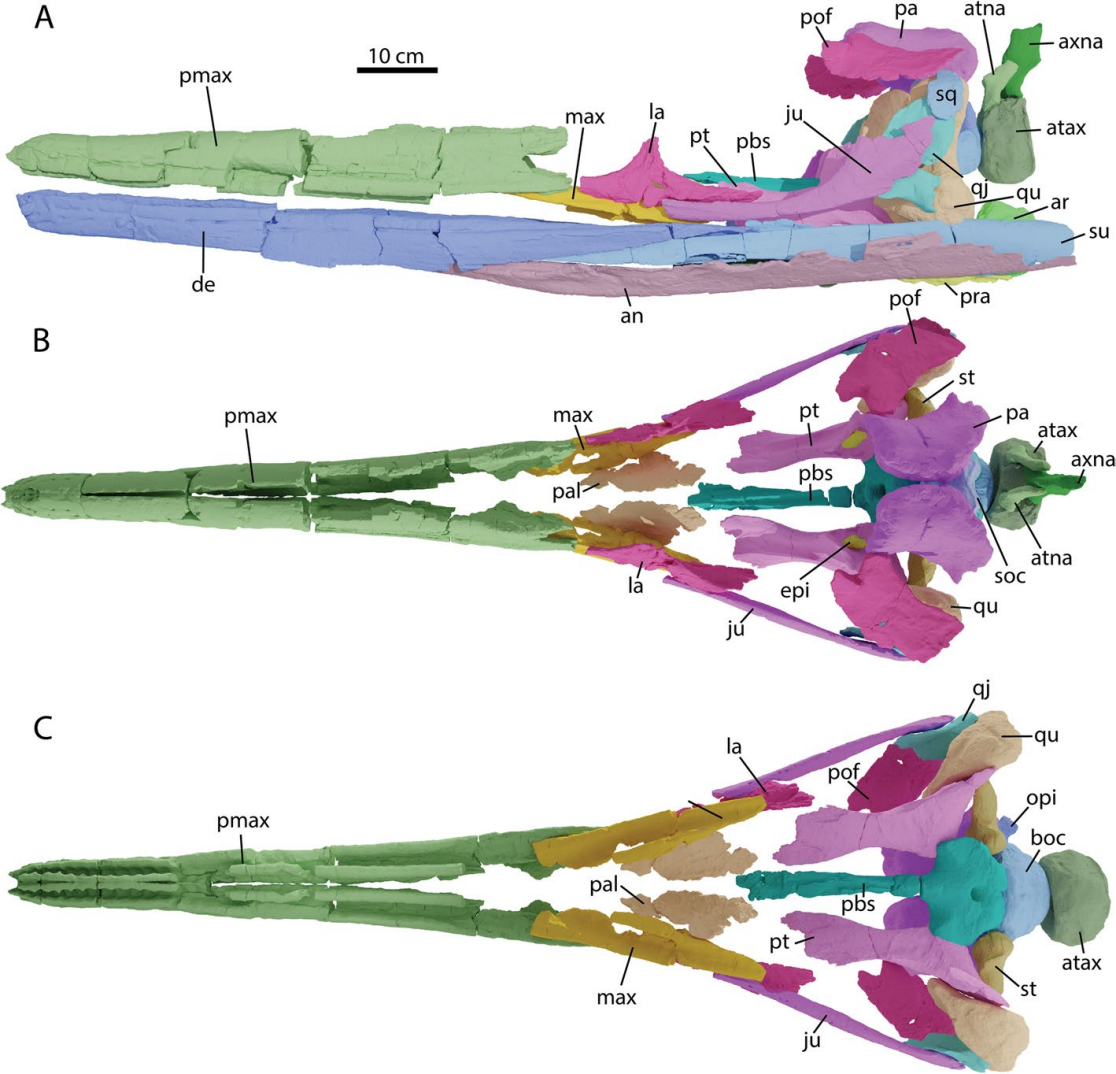
Reconstructed cranium of *Argovisaurus martafernandezi*, holotype PIMUZ A/III 5279 in: **A** lateral view; **B** dorsal view; **C** ventral view. Abbreviations: an, angular; ar, articular; atax, atlas-axis pleurocentrum; atna, atlas neural arch; axna, axis neural arch; boc, basioccipital; de, dentary; epi, epipterygoid; ju, jugal; la, lacrimal; max, maxilla; opi, opisthotic; pa, parietal; pal, palatine; pbs, parabasisphenoid; pmax, premaxilla; pof, postfrontal; pra, prearticular; pt, pterygoid; qj, quadratojugal; qu, quadrate; soc, supraoccipital; sq, squamosal; st, stapes; su, surangular

The material was mechanically prepared by Thomas Imhof and subsequently CT- and surface-scanned, depending on the level of internal detail necessary for the description. Surface scanning was done using the Artec Spider and Artec Eva (Artec Europe, Luxemburg) by the authors. CT-scans were performed with industrial CT-scanning at the Eurofins Qualitech AG, Mägenwil, Switzerland. Beam settings were set dependent on object size and details to be obtained. Meshes of all the surface scans and segmented CT-data are available via Morphosource. CT-data are currently still under study therefore tiff stacks of the CT-data are available upon direct request to the authors. Once the surface models of all cranial elements were made, we used Blender v. 2.91.0–2.93.0 and v. 3.2.0 to model the bones to their in vivo position (Figs. 3, 8 and 10 for 2D image of in vivo rendering). For educational and expositional purposes, an interpretative reconstruction of the entire cranium is being made, using several well-known parvipelvian ichthyosaurs to reconstruct missing elements. This model is therefore an interpretation on what the complete cranium would have looked like. The model will likewise be publicly available upon completion.

Terminological note: There has been discussion over the naming conventions of higher taxonomic clades within Parvipelvia [31–34]. Throughout this manuscript we will follow the recent recommendations by [31]. This means we will refer to the most inclusive clade containing both *Ophthalmosaurus icenicus* and *Platypterygius platydactylus* as Ophthalmosauria (infraorder) instead of Ophthalmosauridae (family).

**Institutional abbreviations:**

**MNHNL**: Muséum national d’histoire naturelle du Luxembourg, Luxembourg-ville, Luxembourg; **PIMUZ**: Department of Palaeontology, University of Zürich, Zürich, Switzerland; **UAMES**: University of Alaska Museum, Fairbanks, USA

**Systematic Paleontology**

Order Ichthyosauria de Blainville, 1835

Infraorder Ophthalmosauria Motani, 1999

*Argovisaurus* genus novum

**Zoobank registration**: act:C3312628-1544-4B87-BBE3-B12346A30BE3

**Etymology**: After the original Latin (and current Italian and Rätoromanic) name of Canton of Aargau: Argovia, where the holotype was found.

**Type species**. *Argovisaurus martafernandezi* gen. et sp. nov. (see below).

**Diagnosis**. As for the type and only species (see below).

### *Argovisaurus martafernandezi*

Figures 1, 3, 4, 5, 7, 8, 10, 9, 11, 12, 13 and 6

**Fig. 4:**
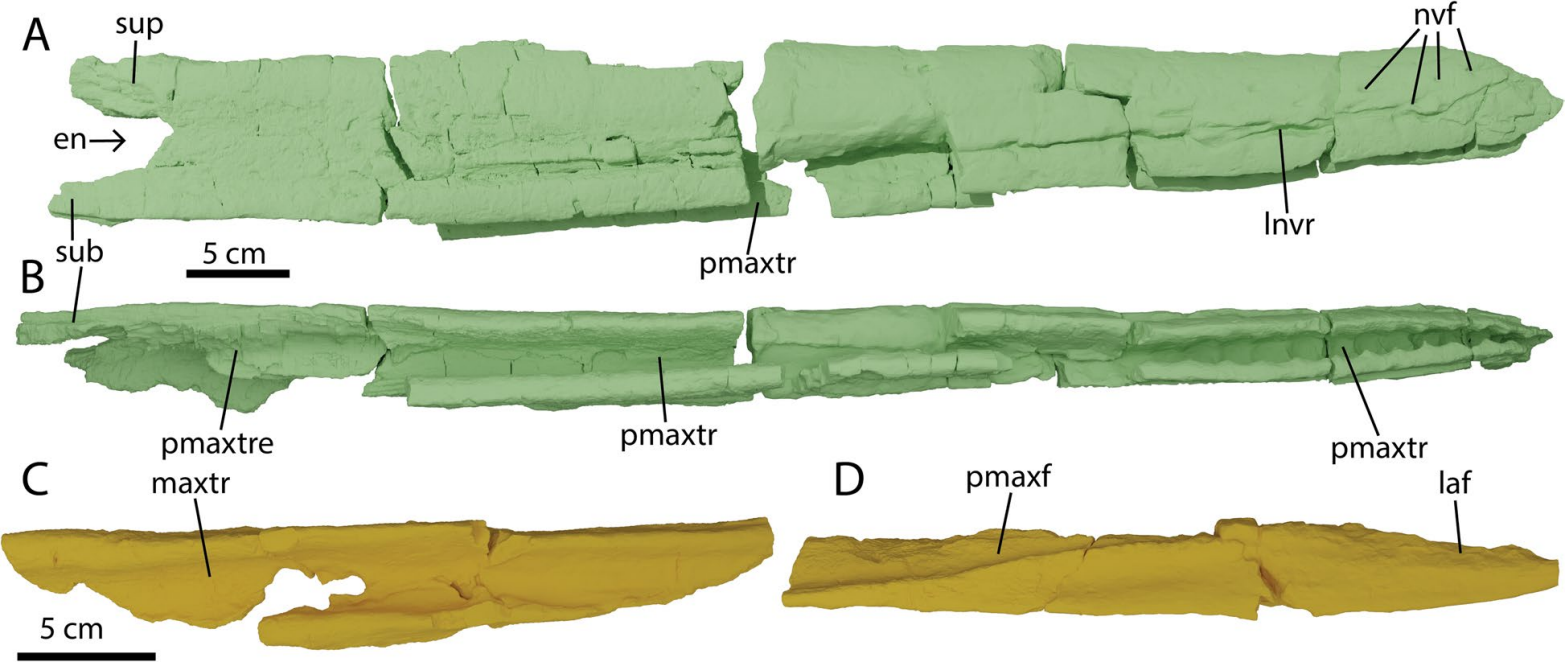
Rostral elements of *Argovisaurus martafernandezi*, holotype PIMUZ A/III 5279. Right premaxilla in: **A** lateral and **B** ventral view. Left maxilla in: **C** ventral and **D** lateral view. Abbreviations: en, external naris; laf, lacrimal facet; lnvr, lateral neurovascular row; maxtr, maxillary tooth row; nvf, neurovascular foramen; pmaxf, premaxilla facet; pmaxtr(e), premaxillary tooth row (posterior ending); sub, subnarial process; sup, supranarial process

**Fig. 5:**
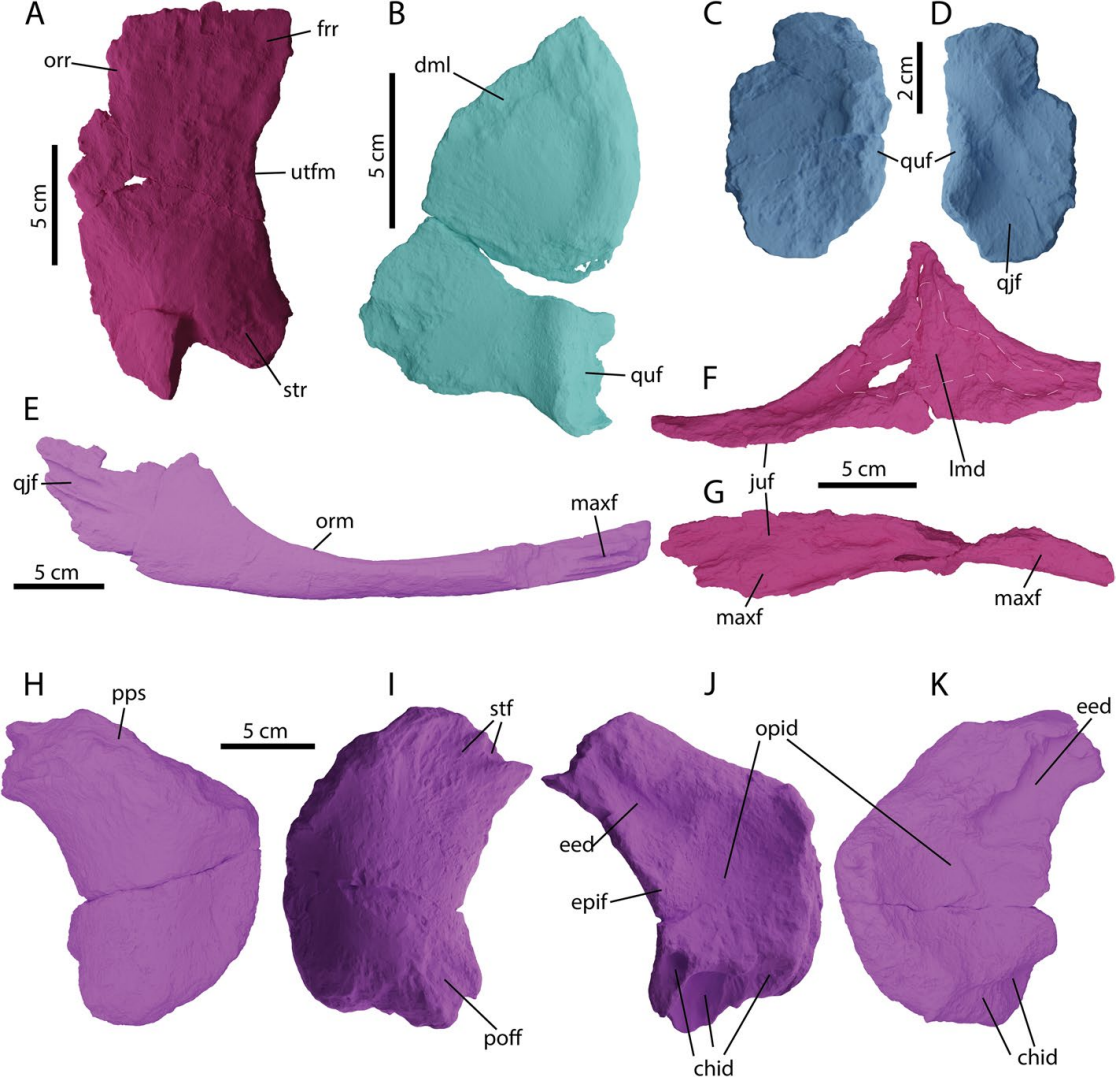
Circumorbital region, cheek region and skull roof elements of *Argovisaurus martafernandezi*, holotype PIMUZ A/III 5279. **A** Left postfrontal in dorsal view; **B** right quadratojugal in medial view; left squamosal in **C** lateral and **D** medial view; **E** left jugal in medial view; left lacrimal in **F** medial and **G** ventral view; right parietal in: **H** dorsal and **K** ventral view; left parietal in: **I** dorsal and **J** ventral view. Abbreviations: chid, cerebral hemisphere indentation; dml, dorsomedial lamina; eed, extra-encephalic depression; epif, epipterygoid facet; frr, frontal ramus; juf, jugal facet; lmd, lacrimal medial depression; maxf, maxilla facet; opid, optic lobe indentation; orm; orbital margin; orr, orbital roof; poff, postfrontal facet; pps, post-parietal shelf; qjf, quadratojugal facet; quf, quadrate facet; stf, supratemporal facet; str, supratemporal ramus; utfm, upper temporal fenestra margin

**Fig. 6:**
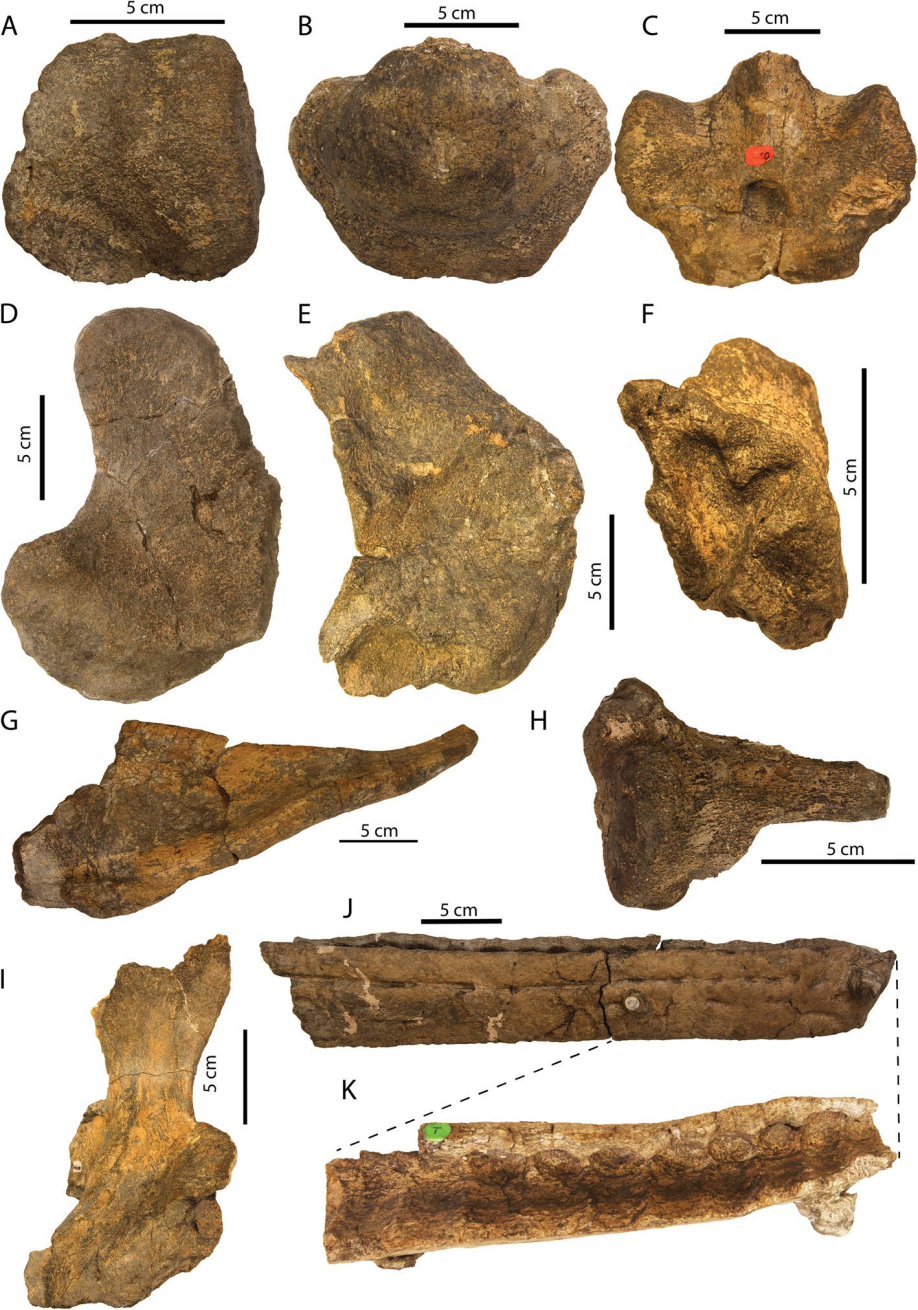
Photos of a selection of cranial and postcranial elements of *Argovisaurus martafernandezi*, holotype PIMUZ A/III 5279. **A** Left articular in medial view. **B** Basioccipital in posterior view. **C** Basisphenoid in ventral view. **D** Right quadrate in posterolateral view. **E** Left parietal in ventral view. **F** Right opisthotic in medial view. **G** Right clavicle in dorsal view. **H** Left parietal in anterior view. **I** Left pterygoid in dorsal view (small portion of quadratojugal still attached). **J** Anterior right dentary in lateral view. **K** Detail of anterior right dentary in dorsal view

**Etymology**. Honouring the renowned paleontologist and ichthyosaur expert Marta Fernández, who has been a pioneer in the topic of marine reptiles and has, among her numerous works, described the rare ichthyosaurs from the Middle Jurassic of South America.

**Holotype and only known specimen:** PIMUZ A/III 5279

**Type locality:** Auenstein quarry at Oberegg, Kanton Aargau, Switzerland

**Type horizon and age:** Lower Acuminata beds of the Hauptrogenstein Formation, (Middle-Late) Bajocian

**Zoobank registration:** act:23C2BD71-8CF0-4D99-848A-0D631518415B

**Diagnosis:**

A large ophthalmosaurian characterized by the following unique combination of characters: **basioccipital** with reduced extracondylar area (shared with *Mollesaurus*); basioccipital peg reduced and not reaching further anteriorly than basisphenoid facet (differs from non-ophthalmosaurian parvipelvians); basioccipital large anterior notochord pit (present but relatively smaller in *Arthropterygius chrisorum*, *Ophthalmosaurus icenicus*) (Fig. 9); **parabasiphenoid** basipterygoid processes projected latero-anteriorly (shared with *Ichthyosaurus*, *Sveltonectes, Baptanodon* and possibly *Mollesaurus*) (Fig. 9); **supraoccipital** antero-posteriorly elongated (possible autapomorphy) (Fig. 11); **stapes** with offset dorsally projected opisthotic facet (possible autapomorphy) (Fig. 12); **quadrate** without distinct occipital lamella (shared with *Stenopterygius*, different from most ophthalmosaurs) and with a small angular process (shared with *Nannopterygius*, *Undorosaurus gorodischensis*, *Arthropterygius volgensis and Palvennia*) (Fig. 12); **premaxilla/dentary** asymmetrically developed, premaxilla with tapering anterior end, dentary with rounded blunt end; both with pseudoalveoli present anteriorly, but not posteriorly (anterior pseudoalveoli shared with *Temnodontosaurus trigonodon*, *O. icenicus*) (Figs. 3, 4 and 8); **premaxilla** with sub- and supranarial processes present and equal in length (shared with *Platypterygius australis*, notably different from *Stenopterygius* and *O. icenicus*) (Fig. 4); **jugal** with little curvature and broad posterodorsal flange (as in *Pl. australis*, but unlike *O. icenicus*, *Ar. lundi*, the referred specimen of *Muiscasaurus catheti* and *Stenopterygius*); **quadratojugal** with quadrate facet posteriorly directed and parallel to the ventral margin (potential autapomorphy) (Figs. 5 and 10); **clavicle** with broad medial sheet and short distal process (shared with *Nannopterygius, Thalassodraco* and *Ar. lundi*) (Fig. 13); **ribs** antero-dorsal ribs with heads connected by a sheet of bone (as in *Eurhinosaurus*, differing from *Stenopterygius* and all ophthalmosaurs which have widely separated rib heads); ribs rounded in cross section with distal grooves in dorsal ribs, similar to *Mollesaurus*, figure-eight shaped in *Stenopterygius, O. icenicus*, and many others) (Fig. 13).

## Description

### General

A large ichthyosaur with a skull estimated at 120 cm in length (Fig. 3A-C). The snout and jaws are robust, the snout is about equal in length the posterior cranium, cheek area is higher than long and relatively short compared to the orbit, maxilla excluded from the ovoidal external naris, pseudoalveoli present anteriorly in the premaxilla and dentary (Fig. 3A-C). Ribheads contain a sheet of bone between the capitulum and tuberculum.

### Dermatocranium

#### Premaxilla

Both premaxillae of PIMUZ A/III 5279 are almost completely preserved (Fig. 4 and data morphosource). Like in all ichthyosauriforms the element forms around 80 percent of the rostrum. Anteriorly the premaxilla is h-shaped in cross section, consisting of the lingual- and labial walls of the alveolar groove and a dorsal ramus. The right and left premaxillae contact each other along the midline anteriorly and diverge around the midpoint of the prenarial rostrum (Fig. 3A-C). The premaxilla only tapers at the very anterior end, but does come to a tapering margin (Fig. 4A). Anteriorly many neurovascular foramina can be seen, possibly carrying CN V (vagus nerve) [35] and associated vasculature (Fig. 4B). No obvious medial vomerine ramus is preserved on either premaxilla, but as the area for the vomerine ramus is relatively poorly preserved in both elements, we cannot rule out one was present. Anteriorly, well-developed pseudoalveoli are present, separated by thickening of the lingual and labial walls of the premaxilla. These depressions fade out posteriorly until they are no longer truly discernable in the alveolar groove after approximately tooth position 15 (Fig. 4B). This condition was previously considered an autapomorphy of *Ophthalmosaurus* [16], but has also been observed in *Temnodontosaurus trigonodon* and occasionally occurs in more derived ophthalmosaurs [36, 37]. Total premaxillary tooth count is difficult to assess, but we estimate between 30–40 teeth were present. Posteriorly the premaxilla contains a supra- and subnarial processes of equal length, a condition shared with *Platypterygius australis* and *Caypullisaurus* [38, 39].

#### Maxilla

Both maxillae of PIMUZ A/III 5279 are preserved. The right maxilla is only half-preserved and still in articulation with the premaxilla (data morphosource). The left maxilla is freely preserved and close to complete (Fig. 4C, D). The maxilla is a shallow, anteroposteriorly elongated triangular structure and does not display a dorsal process, either posterior to or within the narial opening. There is an elongate groove anterolaterally on the dorsal surface which is the premaxillary facet. The lacrimal facet is placed posteriorly on the dorsal surface and is less well delineated. Posteriorly in ventral view, the alveolar groove is visible without discernable tooth impressions. The maxilla likely did not participate in the external naris as reconstructed (Fig. 3A), being excluded by contact between the subnarial process of the premaxilla and the lacrimal. The configuration of the maxilla is unlike *Aegirosaurus*, *Platypterygius australis*, and *Athabascasaurus* in which a dorsoventrally deep maxilla forms the lower edge of the narial opening [38, 40, 41].

#### Jugal

Both jugals of PIMUZ A/III 5279 have been recovered (Fig. 1). The left jugal is slightly better preserved than the right and is therefore figured in detail (Fig. 5E). The jugal is anteroposteriorly straight with a dorsoventrally low, broad postorbital ramus, giving it an overall straight morphology, akin to *U. gorodischensis* and somewhat reminiscent of adult *Stenopterygius* [42, 43], but unlike *Ar. lundi*, *Mollesaurus* and *O. icenicus* in which the dorsal ramus is more slender [15, 16, 44]. Anteromedially at the tip the jugal displays a distinct groove bordered by thick ridges dorsally and ventrally (Fig. 5E). This area is the articulation facet for the lacrimal and maxilla. Likewise, there are similarly distinct grooves bordered by ridges posteromedially on the postorbital ramus, this area is likely the articulation facet for the quadratojugal.

#### Lacrimal

A complete left lacrimal is preserved with PIMUZ A/III 5279 (Figs. 1 and 5F-G). The lacrimal is triangular in outline (Fig. 5F). Centromedially it displays a shallow depression, which is hypothesized to be the location of potential salt glands [45]. Posteriorly, the lacrimal thickens (Fig. 5F). Posterodorsally the lacrimal contains a large smooth surface, which forms the anteroventral margin of the orbit. Posteroventrally a large surface is visible containing facets for the maxilla and jugal. Anteriorly the lacrimal contains a thickened smooth ridge, which forms the posterior margin of the external naris, unlike in *Cryopterygius (*= *Undorosaurus)*? *kristiansenae* and *Platypterygius australis*, in which the lacrimal is excluded from the external nares by the maxilla [38, 43, 46]. The lacrimal does not show any extrusions into the external narial opening such as described in many ichthyosaurs, although the quality of preservation may be partially responsible [47, 48]. Ventrally the lacrimal displays two posteriorly located facets for the jugal (more laterally) and the maxilla (more medially) and one anteriorly located facet for the maxilla (Fig. 5G).

#### Postfrontal

We tentatively identify a right postfrontal (Figs. 3 and 5A). Its shape is a mediolaterally broad semicircle. The posterior end is slightly elevated compared to the anterior end. No clear facets are visible. The anterior head is not as distinct as in other parvipelvians e.g. [38, 42]. The identified postfrontal is dorsoventrally very thin compared to other recovered elements, we are unsure whether this is genuine morphology or a diagenetic artifact.

#### Quadratojugal

The right quadratojugal of PIMUZ A/III 5279 is preserved (Fig. 5B). It is triangular in outline, but dorsoventrally higher than anterioposteriorly long. The anterior margin is straight, whereas the posterior margin is slightly convex. The element thickens slightly towards the posterior as seen in medial view (Fig. 5B). The quadrate facet is set on a thickened ramus and is directed posteroventrally as reconstructed (Figs. 3A and 10D). A posteroventrally directed quadrate facet seems the norm in all parvipelvians. However, there is a distinct difference between taxa where the facet is placed on the element. PIMUZ A/III 5279 is more similar to *Stenopterygius*, *O. icenicus* and possibly *Mollesaurus* as its quadrate facet is in line with the posterior margin of the element rather than to *Cr. kristiansenae* and more derived platypterygiines in which the facet is more in line with the ventral edge [15, 16, 33, 38, 42, 43, 49]. The quadrate process is dorsoventrally and proximodistally in line with the dorsal body of the quadratojugal and is therefore not really offset as in e.g. *Ichthyosaurus* [50]. There is extensive variation among ophthalmosaurian quadratojugals related to relative length of the cheek, with the exposed dorsal ramus of those of large platypterygiines (e.g., *Platypterygius australis*, *Kyhytysuka*; [33, 38]) being wide (the body of the quadratojugal as long as or longer than tall), square, and large, forming an extensive portion of the cheek region. In many more early diverging taxa (*O. icenicus*, *Thalassodraco*) the dorsal ramus of the quadratojugal is narrow (much taller than long), almost triangular with the vertex oriented dorsally, and playing a minor role in the cheek region [16, 51]. PIMUZ A/III 5279 shows an intermediate morphology in this regard, having a relatively broad dorsal region, but it remains taller than wide.

#### Squamosal

We tentatively identify a partial left squamosal in PIMUZ A/III 5279 (Figs. 3A, 5C, D and 10B-D). There is a small broken element consisting of a sheet of bone and a semicircular thickened process. The process is interpreted as the quadrate facet of the squamosal, making the sheet of bone the partial anterior and ventral projections of the squamosal. Medially a small triangular facet is visible, likely holding the dorsal margin of the quadratojugal (Fig. 5D) The squamosal is present in early-diverging opthalmosaurids, but is often lost in platypterygiines (e.g., *Platypterygius austalis*: [38]).

#### Parietal

Both parietals were recovered from PIMUZ A/III 5279 (Fig. 5H-K). The parietal is robust, being almost as mediolaterally wide as anteroposteriorly long and semilunate in shape (Fig. 5H-K). The supratemporal ramus is relatively short and not distinctly offset from the anterior part of the element. This morphology is quite similar to *Stenopterygius* (except *S. aaleniensis*) [13, 42]. In contrast *O. icenicus, Palvennia* (= *Arthropterygius*?) *hoybergeti and Nannopterygius* have a distinct offset supratemporal ramus [16, 52, 53], or an elongated ramus as in *Thalassodraco* [51]. The antimeric parietal facet (medial edge) is rounded in dorsal view, as in *Stenopterygius* and not flattened as in e.g. *Cr. kristiansenae* and *Nannopterygius* [42, 43, 53]. There are some noteworthy differences between the left and right parietals of PIMUZ A/III 5279 (Fig. 5H-K). The first is the presence of distinct post-parietal shelf (as seen in dorsal view) on the right element (Fig. 5H) and the absence of this morphology on the left element (Fig. 5I). Ventrally the left and right parietals are also slightly different (Fig. 5J-K). Both left and right elements display an epipterygoid facet, but the in left element the facet is much more pronounced (Fig. 5J). Moreover, the left element shows a deep ventrolateral indentation (interpreted as the indentation for the cerebral hemisphere (sensu Zverkov & Efimov, 2019), which is largely absent in the right element (Fig. 5K). The right element does show a deepened posterolateral extra-encephalic depression compared to the left element (Fig. 5K). Both elements do show a large central depression on the ventral side, which is the large indentation for the optic lobe [54].

### Dermatocranium: palate

#### Pterygoid

**Fig. 7:**
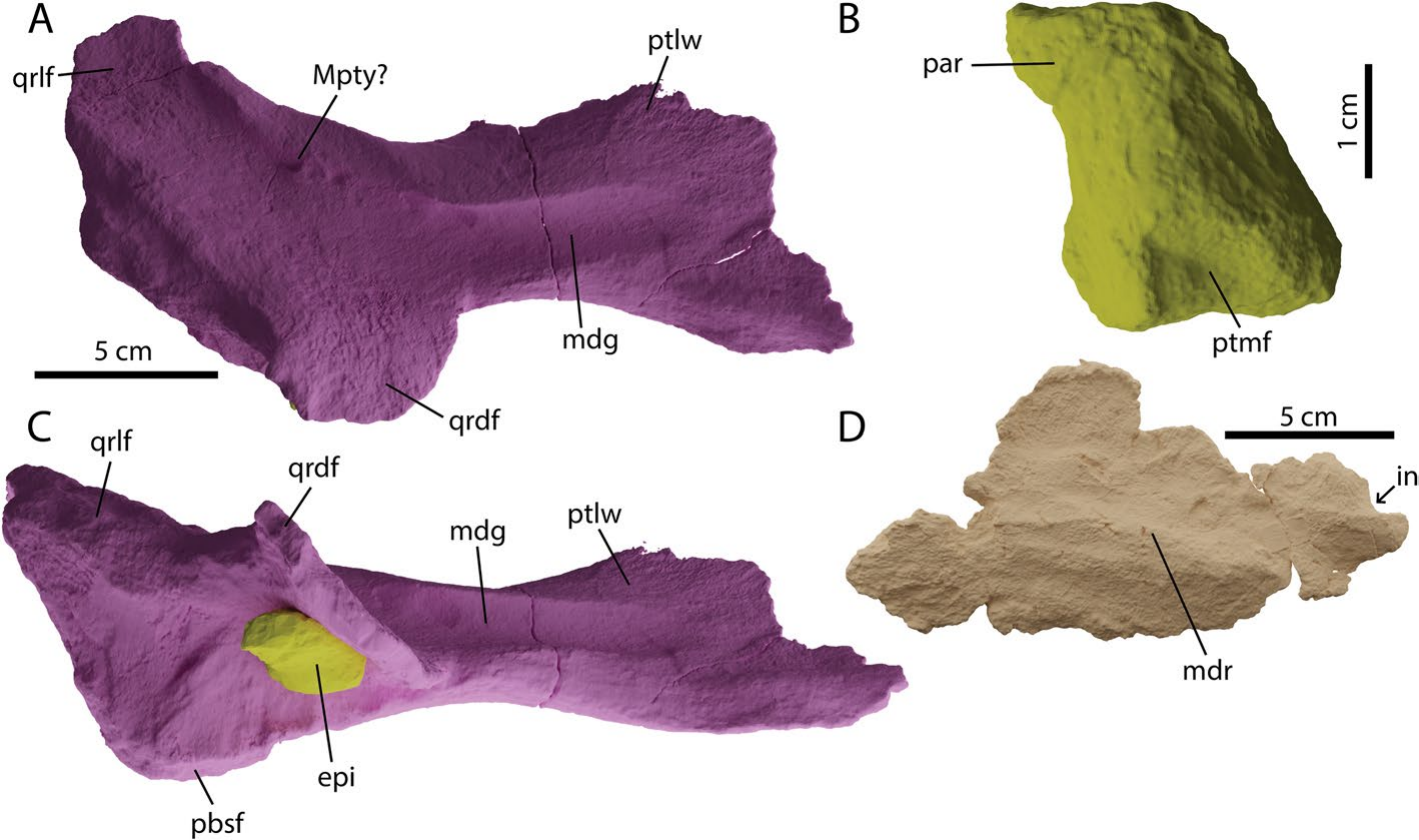
Palatal elements of *Argovisaurus martafernandezi*, holotype PIMUZ A/III 5279. Left pterygoid in: **A** laterodorsal and **C** mediodorsal view; **B** right epipterygoid in lateral view; **D** left palatine in laterodorsal view. Abbreviations: epi, epipterygoid; mdg, median dorsal groove; mdr, median dorsal rige; Mpty, insertion area of the pterygoideus muscle; par, parietal ramus; pbsf, parabasiphenoid facet; ptlw, pterygoid lateral wing; ptmf, medial pterygoid facet; qrdf, quadrate ramus dorsal flange; qrlf, quadrate ramus lateral flange

Both pterygoids were recovered with PIMUZ A/III 5279, unfortunately, neither of them complete anteriorly. The left pterygoid is slightly better preserved and thereby used in the reconstruction (Figs. 3 and 10). The pterygoid consists of a wide posterior triradiate quadrate ramus and an anterior sheet of bone representing the palatal ramus (Fig. 7A, C). The palatal ramus is posteriorly constricted before widening anteriorly, a feature generally seen in parvipelvians but different from at least one specimen attributed to *Brachypterygius (*= *Grendelius?)extremus* [49]. In dorsolateral view there is an extended groove bordered by two ridges running along the palatal ramus and ending on the dorsolateral side of the quadrate ramus. This feature is seen in other ophthalmosaurians such as *Sveltonectes* and *Pl. australis* but the morphology is more extremely delineated in PIMUZ A/III 5279 [38, 55]. The quadrate ramus consists of a dorsally projected flange, a medially projected flange containing the basisphenoid facet, and a ventrolaterally projected flange which holds the ventromedial side of the quadrate (Fig. 10A-D). Anterodorsal on the ventrolateral flange small semilunar depressions are visible. We interpret these as the insertion location of the pterygoideus muscle (Musculus adductor internus pterygoideus sensu [50, 56]). The dorsal flange is curved, projecting slightly medially. Both the pterygoids and the palatines are reconstructed to be at a 35–45-degree angle in vivo (Figs. 3 and 10). This results in a parabolically shaped palate. This may seem extreme but is consistent with articulated morphology as seen in *Ichthyosaurus, Pl. australis* and *Temnodontosaurus zetlandicus* [38, 50, 57]. Although there is no clear facet, we tentatively place the epipterygoid anteromedially on between the medial and dorsal flanges of the quadrate ramus. In this way the epipterygoid can contact the prootic and parietal as is normal in reptiles e.g. [58, 59].

#### Palatine

We tentatively identify a right palatine preserved with PIMUZ A/III 5279 (Fig. 7D). It is an elongated elliptical element, being wider in the midsection than at the anterior and posterior margins. Dorsally it displays an anteroposteriorly oriented ridge. Anteriorly, a small invagination is visible, bordered by a small process dorsomedially (Fig. 7D). This invagination would form the posterior margin of the internal naris. Palatines are relatively understudied in ichthyosaurs as they are often obscured or taphonomically lost. However, the overall outline is similar to other non-platypterygiine parvipelvians such as *Ichthyosaurus* and *Stenopterygius* [42, 50], but differs from *Pl. australis*, in which the palatine does not contribute to the posteromedial wall of the internal narial opening [38].

### Dermatocranium: hemimandible

#### Dentary

**Fig. 8:**
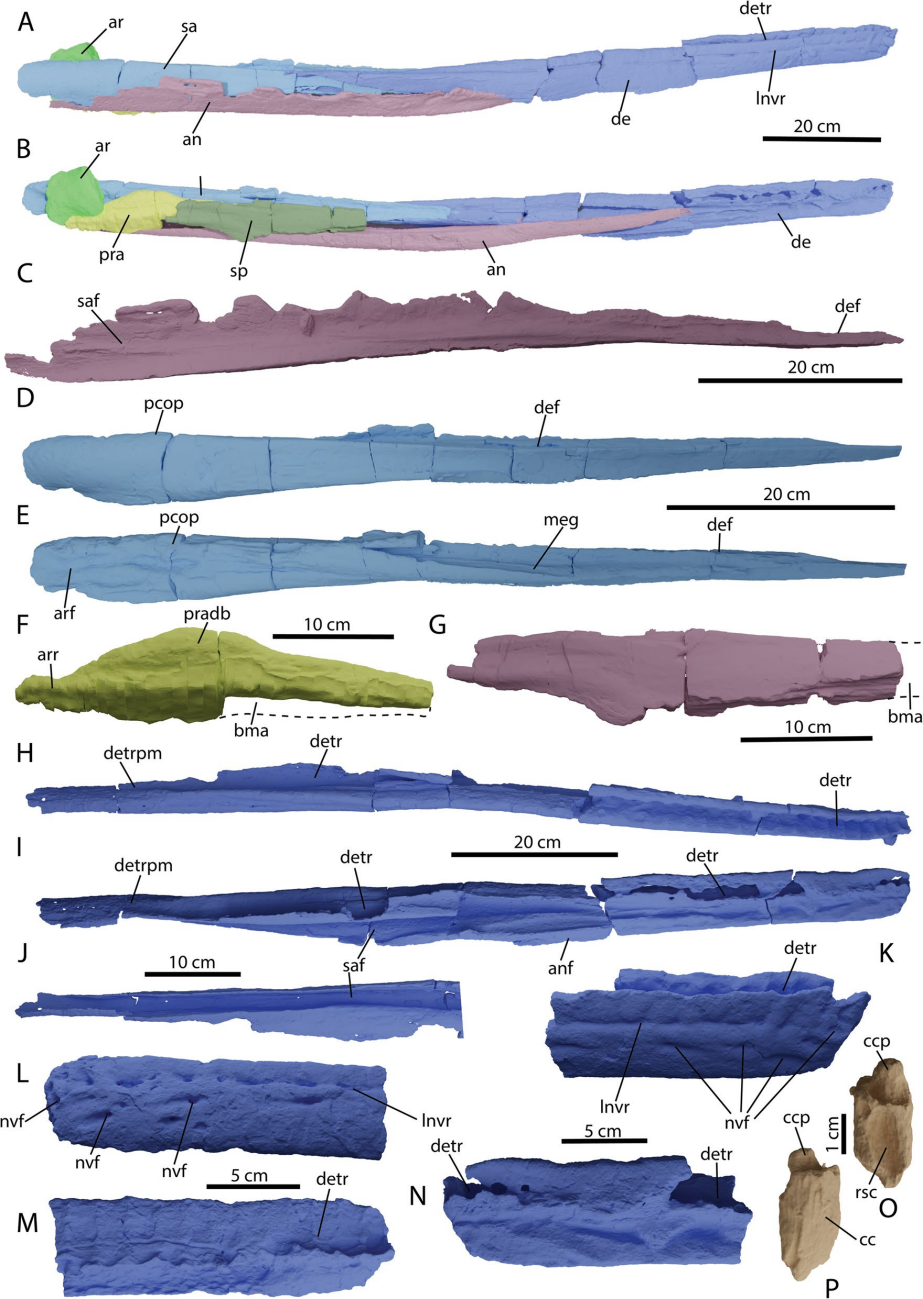
Reconstruction of the right hemimandible in: **A** lateral and **B** medial view (image mirrored), isolated elements and details of either hemimandible (**C**-**N**) and the most complete preserved tooth root of *Argovisaurus martafernandezi*, holotype PIMUZ A/III 5279 (**O**, **P**). **C** Left angular in medial view; right surangular in: **D** lateral and **E** medial view; **F** right partial prearticular in medial view; **G** right partial splenial in medial view; right dentary in: **H** dorsal and **I** medial view; **J** posterior portion of right dentary in ventral view; tip of right dentary in: **K** lateral and **N** medial view; tip of the left dentary in: **L** lateral and **M** medial view; tooth root in: **O** posterior and **P** anterior view. Abbreviations: an, angular; anf, angular facet; ar, articular; arr, articular ramus; bma, broken margin; cc, cellular cementum; ccp, pulpal cementum; de, dentary tooth row; def, dentary facet; detr, dentary tooth row; detrpm; dentary tooth row posterior margin; lnvr, lateral neurovascular row; meg, meckellian groove; nvf, neurovascular foramen; pcop, paracoronoid process; pra, prearticular; pradb, prearticular dorsal bulge; rsc, resorption cavity; sa, surangular; saf, surangular facet; sp, splenial

Both dentaries were recovered with specimen PIMUZ A/III 5279 (Fig. 1). The left dentary is compacted by the premaxillae and the left surangular (data morphosource). Moreover, its anterior tip is slighty pathological (Fig. 8L-M). We will therefore focus on the isolated right dentary for anatomical description (Fig. 8A, B, H–K, N). The dentary forms the largest part of the anterior mandibular ramus (Fig. 8A, B). Like the premaxilla, the dentary displays distinct alveolar depressions (pseudoalveoli sensu [36]) bordered by ridges anteriorly, which fade out until no distinct alveolar depressions are discernible after around tooth position 18 (Fig. 8H). More posteriorly, the dental groove continues along most of the dorsomedial portion of the dentary. As in the premaxilla it is difficult to estimate the complete tooth position count, our estimations lie between 45 and 55. The anterior tip of the dentary is blunt and rounded (Fig. 8K, N). Laterally it contains multiple neurovascular foramina, which are aligned dorsolaterally after tooth position 4 (Fig. 8A). This lateral row of neurovascular foramina continues along roughly 2/3 of the length of the dentary. The (ventro)lingual wall of the alveolar groove is thin and often eroded away, whereas the labial wall and dorsolingual wall are continuously thick (Fig. 8H, I). Anteromedially, two distinct grooves are visible, divided by a ridge. These grooves merge more posteriorly to form the surangular- and likely also the splenial facet (Fig. 8I).

#### Surangular

Both the right and left surangular were recovered with PIMUZ A/III 5279 (Fig. 1). We describe the morphology largely based on the right element as the left is heavily affected by pathologies (see section on pathologies and Fig. 13M, N). The surangular is the thickest element in the mandibular ramus and runs along most of its posterolateral side (Fig. 8A). Anteriorly it tapers to a sharp point (Fig. 8D). A flat facet for the dentary runs along the anterodorsal margin of the element. Posteromedially, it displays some grooves where the articular and prearticular would have articulated (Fig. 8E). Medially, a thickened ridge appears on the midline of the element which projects anterodorsally; anteriorly it forms the side of the Meckelian canal. No distinct preglenoid process (sensu: [17]) was observed; the element does display a small paracoronoid process, but this is definitely not as pronounced as most other parvipelvian ichthyosaurs [16, 38, 42, 50, 54]. It is uncertain whether this is genuine morphology as the element appears weathered in this area. No surangular fossa or foramen is present, in contrast to most other parvipelvians [16, 38, 42].

#### Angular

Both angulars are preserved with PIMUZ A/III 5279 (Fig. 1). They are elongated elements. The dorsoventrally highest part is located approximately posteriorly, after which the element tapers towards the anterior. The angular reaches far up the ventral side of the mandibular ramus (Fig. 8A, B). Medially large grooves are visible, which correspond to the surangular facet (Fig. 8C). Given the large posterior dorsoventral height, it is likely that the angular was extensively exposed compared to the surangular (Fig. 8A). However, since the specimen was not found in articulation, we therefore state this with caution.

#### Prearticular

A partial prearticular was identified with the right mandibular ramus (Fig. 8B, F). The prearticular is an elongated element that contacts the articular posteriorly, the surangular medially and the splenial anterolaterally (Fig. 8B). The dorsoventrally highest area of the prearticular is located centrally, after which the element tapers in both anterior and posterior directions (Fig. 8F). This thickened area potentially also contributed to the glenoid fossa. However, its exact contribution is uncertain.

#### Splenial

We tentatively identify a partial posterior splenial with the right mandibular ramus (Fig. 8B, G). The element is mediolaterally flat and anteroposteriorly elongate. The distinct anterior processes are lost.

#### Missing dermatocranium

Taphonomically lost, or at least not recovered in the excavations, were the following elements of the dermal skull: nasal, frontal, supratemporal, postorbital, prefrontal and vomer.

### Chondrocranium

#### Parabasisphenoid

**Fig. 9:**
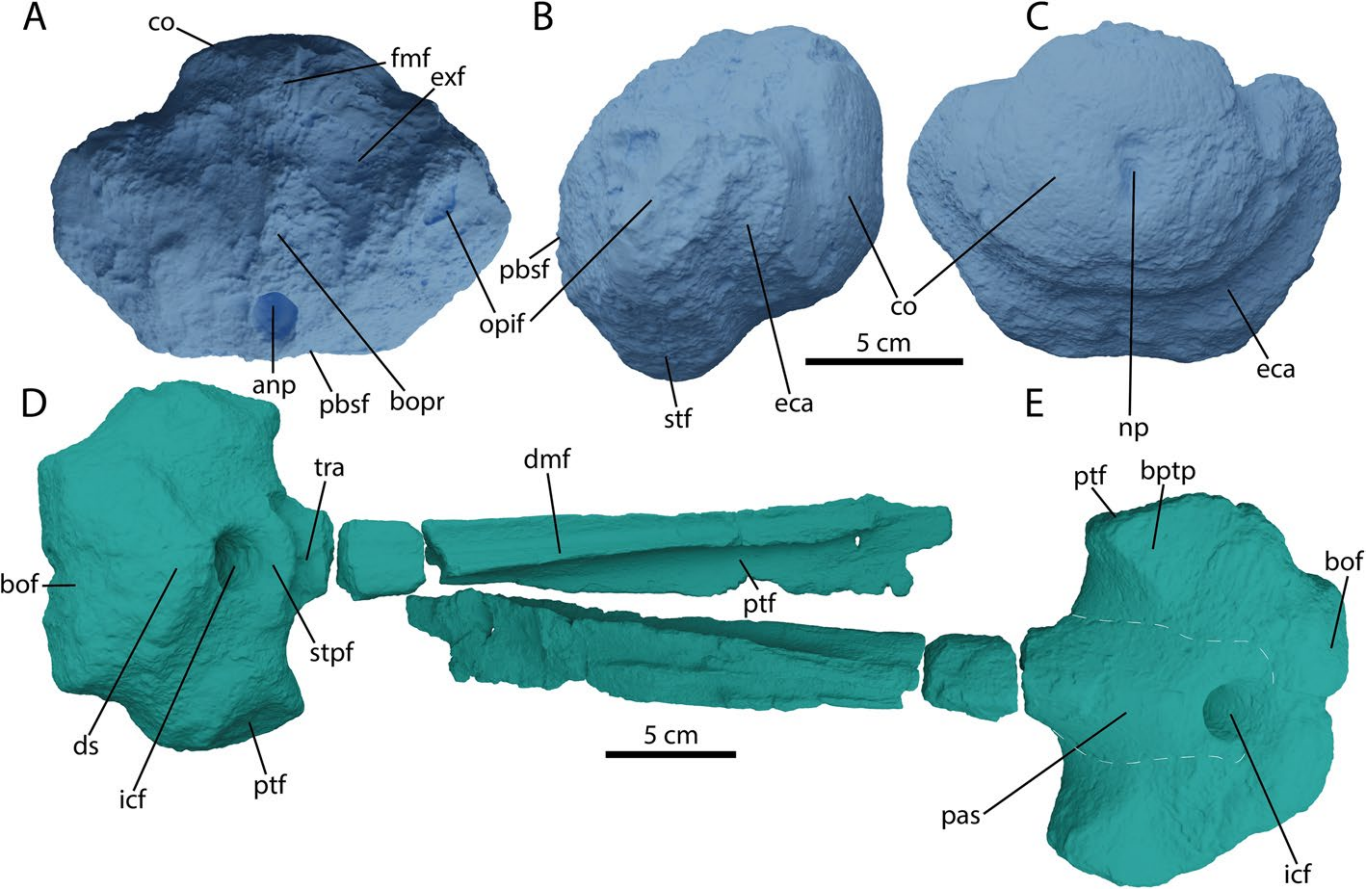
Ventral chondrocranium of *Argovisaurus martafernandezi*, holotype PIMUZ A/III 5279. Basioccipital in: **A** dorsal, **B** right lateral and **C** posterior view. Parabasisphenoid in: **D** dorsal and **E** ventral view. Abbreviations: anp, anterior notochord pit; bof, basioccipital facet; bopr, basioccipital peg region; bptp, basipterygoid process; co, condyle; eca, extracondylar area; dmf, dorsomedian furrow; ds, dorsum sellae; exf, exoccipital facet; fmf, foramen magnum floor; icf, internal coratid foramen; opif, opishtotic facet; pas, parasphenoid; pbsf, parabasisphenoid facet; ptf, pterygoid facet; stf, stapes facet; stpf, sella turcica/pineal fossa; tra, trabecular area

The parabasisphenoid of PIMUZ A/III 5279 is relatively quadrangular in outline in dorsal and ventral view (Fig. 9D, E). The parasphenoid-basisphenoid suture is visible in ventral view, whereby the parasphenoid reaches the anterior margin of the internal carotid foramen (icf) and thereby does not divide the icf ventrally into two separate foramina as in *Temnodontosaurus trigonodon* [60]. An unpaired icf is plesiomorphic for Ophthalmosauria, and in adult *Stenopterygius* it is also observed, although a partial internal separation of the icf is present in immature stages [4, 16, 61]. The internal carotid foramen is centrally placed on the ventral side of the basisphenoid in PIMUZ A/III 5279, differing from *Arthropterygius volgensis*, *Janusaurus lundi* and many derived platypterygiines (e.g., *Br. extremus, Pl. australis, Sveltonectes, Sisteronia*) [38, 44, 49, 62–64]. In ventral view, the basipterygoid processes in PIMUZ A/III 5279 are directed latero-anteriorly. In many ophthalmosaurians they are directed strictly laterally (e.g., *Brachypterygius extremus*, *U. gorodischensis* [43, 63]). However, the condition observed in PIMUZ A/III 5279 is similar to the condition in *Stenopterygius, Baptanodon, Sveltonectes,* and *Ichthyosaurus* [50, 55, 61, 65]. The dorsum sellae and trabecular area are as in most other ichthyosaurs in morphology. There is no apparent stapedial facet on the parabasisphenoid in lateral view. In dorsal view, the basioccipital facet is oriented posteriorly, such that it is small relative to the dorsal plateau (sensu [64]). A notochordal groove runs posteriorly from the anterior dorsal plateau (Fig. 9D, E).

#### Basioccipital

The basioccipital of PIMUZ A/III 5279 is the posterior-most element in the braincase and connects the cranium to the vertebral column. An extensive extracondylar area (eca) can be seen in posterior view (Fig. 9C). The eca is reduced relative to non-ophthalmosaurian baracromians, being similar in extent to condylar exposure in lateral view. Unlike in *O. icenicus*, the cortical bone on the ventral eca is not separated by a notch, but is reduced to a narrow strip [16]. The ventral extent of the eca is greatly reduced relative to non-ophthalmosaurian baracromians such as in *Stenopterygius* and *Chacaicosaurus* [12, 16, 61]. The morphology of the eca is overall very similar to *Mollesaurus* or *Baptanodon* [11, 65]. The condyle bears a dorsoventrally elongated notochord pit (Fig. 9C). In dorsal view, the opisthotic facets are visible anterolaterally and the exoccipital facets posteromedially (Fig. 9A). In addition to those facets, eroded remnants of the foramen magnum floor and much reduced basioccipital peg are visible (Fig. 9A). The basioccipital peg does not reach the anterior edge of the basioccipital, similar to most ophthalmosaurs with the exception of *Arthropterygius chrisorum* [66]. Anteroventral to the basioccipital peg there is a large anterior notochord pit (Fig. 9A). This structure is found in *Baptanodon*, the holotype of *Ar. chrisorum,* and a specimen referred to *Muiscasaurus* among adult parvipelvians, and is seen in fetal stages of *Stenopterygius* [61, 65–67]. No anterior notochordal groove is present. In lateral view, a large facet for the stapes is visible anteroventral to the opisthotic facet (Fig. 9B). In dorsal view the floor of the foramen magnum is not raised more dorsally than the main body of the basioccipital and the floor is not well delineated. A raised delineated foramen magnum floor is seen in *Mollesaurus* [15].

#### Exoccipital

**Fig. 10:**
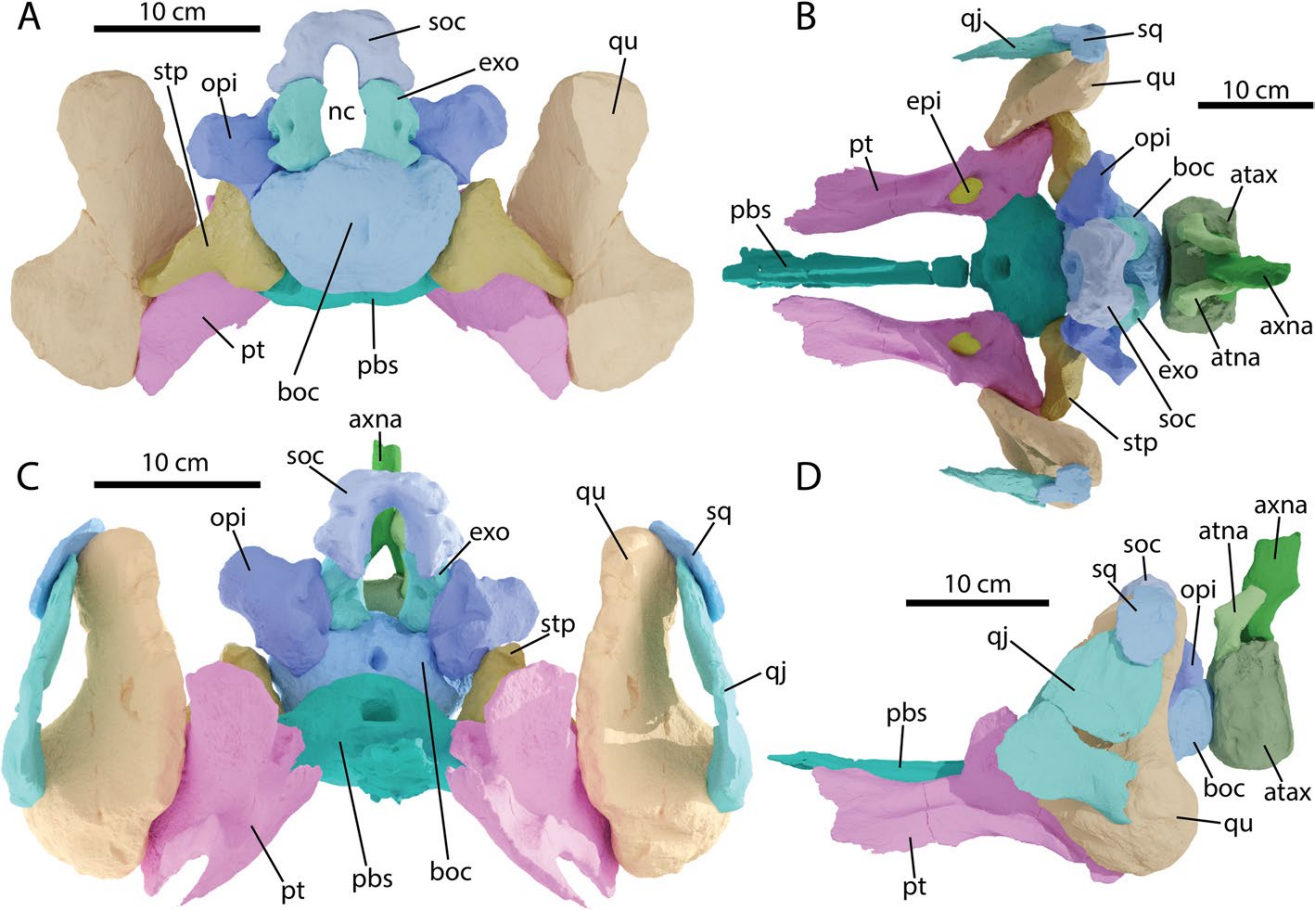
Details of the reconstruction of the braincase and posterior palatal and cheek regions of *Argovisaurus martafernandezi*, holotype PIMUZ A/III 5279 in **A** posterior; **B** dorsal; **C** anterior and **D** left lateral view. Abbreviations: atax, atlas-axis pleurocentrum; atna, atlas neural arch; axna, axis neural arch; boc, basioccipital; epi, epipterygoid; exo, exoccipital; nc, neural canal; opi, opisthotic; pbs, parabasisphenoid; pt, pterygoid; qj, quadratojugal; qu, quadrate; soc, supraoccipital; sq, squamosal; stp, stapes

**Fig. 11:**
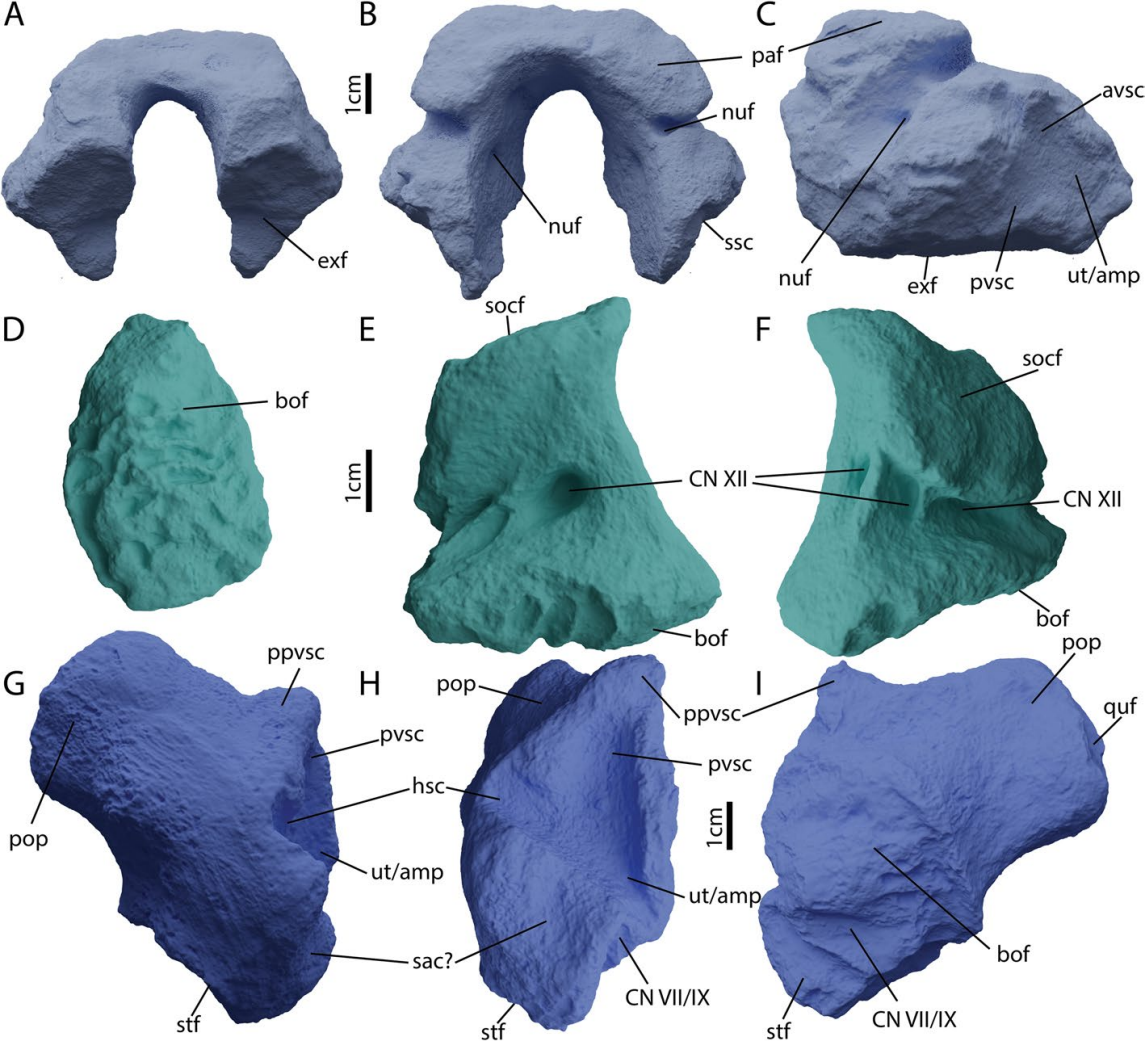
Dorsal chondrocranium of *Argovisaurus martafernandezi*, holotype PIMUZ A/III 5279. Supraoccipital in: **A** posteroventral, **B** anterior and **C** right lateral view. Left exoccipital in: **D** ventral view. Right exoccipital in: **E** medial and **F** posterolateral view. Right opisthotic in: **G** anterior, **H** medial and **I** posterior view. Abbreviations: avsc, anterior vertical semicircular canal indentation; bof, basioccipital facet; CN VII/IX, groove for cranial nerve VII (facial) or IX (accessory); CN XII, hypoglossal foramen (cranial nerve XII); exf, exoccipital facet; hsc, horizontal semicircular canal indentation; nuf, nutritive foramen; paf, parietal facet; pop, paroccipital process; ppvsc, indentation of the external process of the posterior vertical semicircular canal; pvsc, posterior vertical semicircular indentation; quf, quadrate facet; sac, sacculus indentation; socf, supraoccipital facet; ssc, semicircular canal indentation; stf, stapes facet; ut/amp; utriculus and/or ampulus indentation

Both exoccipitals were recovered from PIMUZ A/III 5279. Only the right one is fully preserved and used in the reconstruction (Fig. 10). The exoccipital has an anteriorly sloping triangular outline in medial view and is columnar in posterior view (Fig. 11E, F). It is relatively high compared to the descending rami of the supraoccipital and thereby contributes around half of the height of the margin of the foramen magnum (Fig. 10). This contrasts with *O. icenicus* but is similar to *Pl. australis* and *Baptanodon* [16, 38, 65]. The exoccipital remains tall as in *Baptanodon* and *O. icenicus* rather than squat as in *Acamptonectes* [16, 32, 65]. The ventrally directed basioccipital facet is very rugose and contains grooves, potentially indicating a larger cartilaginous contact between the exoccipital and the basioccipital (Fig. 11D). The dorsally projected supraoccipital facet on the contrary is smooth and remains undivided unlike in *Sveltonectes* [55]. The exoccipital shows many potential entrances and exits for the hypoglossal nerve (CNXII) (and indeed there could have been multiple branches; this varies intraspecifically in *O. icenicus* [68]). Medially there is a very large foramen centrally placed on the element. Anterior to this foramen are two minor more elongated foramina divided by a thin sheet of bone. Posterolaterally two foramina can be seen. The more medial of the two is positioned laterally near the midpoint of the element (in direct posterior view). The two foramina are divided by a thick sheet of bone, which likewise forms the posterolateral margin of the exoccipital.

#### Opisthotic

Both opisthotics of PIMUZ A/III 5279 are well-preserved. The opisthotic consists of a short, stout paroccipital process and a broad medial head containing the basioccipital and stapedial facets and the indentations for the semicircular canals (Fig. 11G-I). In posterior view, a large surface is visible directed posteroventrally. The surface is well delineated dorsally and laterally and is made up of the posteroventrally directed basioccipital facet and at the ventral margin the stapes facet. There is a shallow groove visible just dorsal to the posterior extension of the indentation for the utriculus or ampulla. This groove possibly held the vagus nerve. As visible in ventral view, the basioccipital- and stapes facets are separated by a shallow groove, which likely held either the hyomandibular branch of the facial nerve (cranial nerve VII) or the glossopharyngeal nerve (cranial nerve XI). In medial view, the indentations for the semicircular canals are present. The indentation is distinctly V-shaped, with the angle between the impression of the posterior vertical semicircular canal (pvsc) and the horizontal semicircular canal (hsc) being much less than 90°. The impression for the pvsc is distinctly thicker than the indentation for the hsc. As the pvsc extends relatively far dorsally, a dorsally projected process is formed as is also seen in *Stenopterygius* [61]. The indentation area for the sacculus, utriculus and ampulla is very limited. There is a small depression visible just ventral to the main indentations of the semicircular canals. It is possible that this depression partially holds the sacculus given the limited space in the main indentation (Fig. 11H). The v-shaped morphology is common in ophthalmosaurians with some notable exceptions in which the impression of the sacculus is expanded (*Pl. australis, Sisteronia* and *Acamptonectes*, which has a distinct ventral bulge holding the sacculus [32, 38, 64]).

#### Supraoccipital

The supraoccipital of PIMUZ A/III 5279 is quadrangular in outline in posterior view (Fig. 11A). This outline is mainly defined by the relatively large lateral parietal facets (Fig. 11A, B). In most parvipelvians the supraoccipital is dorsally more rounded in outline, e.g. *O. icenicus*, *Stenopterygius* and *Ar. hoybergeti* [16, 61, 62]; *Acamptonectes* comes closest in quadrangular shape [32]. The supraoccipital contributes substantially to the foramen magnum, roughly 50% of the height of the foramen. This is similar to most ophthalmosaurs, e.g. *Pl. australis*, *Ar. hoybergeti*, *O. icenicus* and *Nannopterygius* [16, 38, 52, 53], but differs from more basal parvipelvians [50, 69], and is somewhat ontogenetically variable in *Stenopterygius* [61]. In anterior view, depressions of putative nutritive foramina are visible asymmetrically divided between the left and right sides (Fig. 11B). Nutritive foramina are sporadically present in euichthyosaurs throughout phylogeny, e.g. *Ichthyosaurus*, *Temnodontosaurus*, *Acamptonectes* and *O. icenicus* [16, 32, 50, 69]. The most striking feature of the supraoccipital of PIMUZ A/III 5279 is the anteroposterior elongation of the exoccipital processes, seen in lateral and ventral view (Fig. 11A, C). Such elongation is not seen in any euichthyosaur to our knowledge. The otic capsule impression is directed anterolaterally and shows a triradiate morphology, different from some ophthalmosaurians such as *Sisteronia,* and *Pl. hercynicus* which show an almost lunate capsular impression [32], 2014), but similar to e.g. *Pl. australis* [38]. The exoccipital facets are large and directed entirely ventrally (Fig. 11A). The exoccipital processes curve slightly medially towards their ventral ends, forming a key-hole shaped foramen magnum, as in *Ophthalmosaurus icenicus* and *Baptanodon natans* [16, 65].

#### Prootic

Neither prootic was recovered.

### Splanchnocranium

#### Stapes

**Fig. 12:**
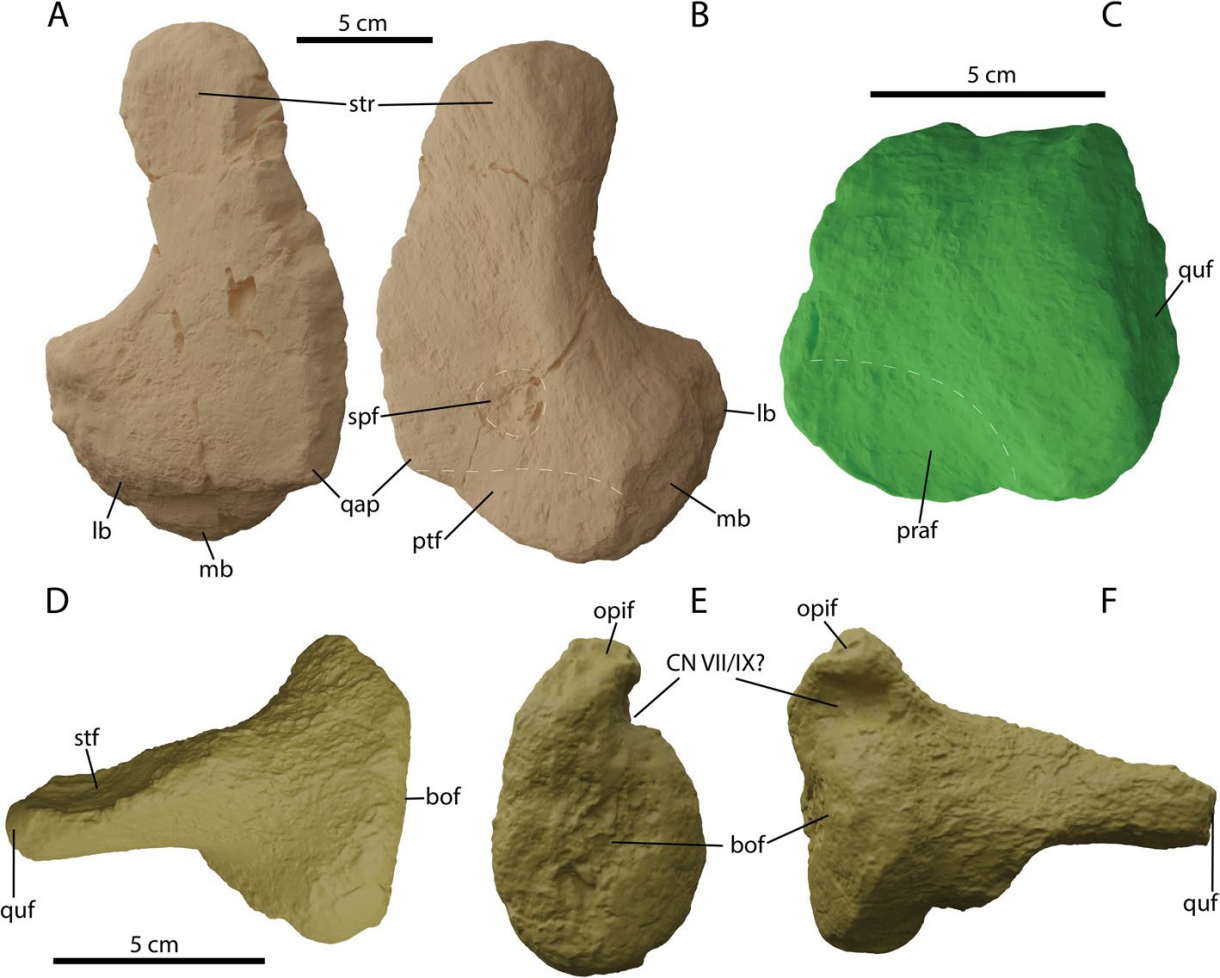
Splanchnocranial elements of *Argovisaurus martafernandezi*, holotype PIMUZ A/III 5279. Right quadrate in: **A** anterolateral and **B** posteromedial view. Left articular in medial view **C**. Left stapes in: **D** posterior, **E** medial, **F** anterior view. Abbreviations: bof, basioccipital facet; CN VII/IX, groove for cranial nerve VII (facial) or IX (accessory); lb, lateral boss; mb, medial boss; opif, opisthotic facet; praf, prearticular facet; ptf, pterygoid facet; qap, quadrate angular process; quf, quadrate facet; spf, stapes facet; stf, supratemporal facet; str, supratemporal ramus

Both stapes of PIMUZ A/III 5279 are preserved, the left element in the better condition and is thereby figured and used in the reconstruction (Fig. 12D-F). The stapes consists of an elongated lateral process and a large medial head (Fig. 12D-F). The quadrate facet is not enlarged as in e.g., *Cr. (*= *Undorosaurus)*? *kristiansenae, Grendelius alekseevi*, *Janusaurus lundi* and *Sisteronia* [44, 52, 62–64, 70], but tapers laterally as in *Stenopterygius* [61]. On the stapedial shaft, a distinct facet is visible, which likely contacted the descending ramus of the supratemporal. There is a distinct torque in the stapedial shaft best visible medial to the supratemporal facet and lateral to the medial head (Fig. 12D, F and data morphosource). This condition is somewhat reminiscent of *O. icenicus* [16]. Medially, the stapes contains two facets, a large round basioccipital facet ventrally and a smaller ellipsoidal opisthotic facet dorsally (Fig. 12F). Between the opisthotic and basioccipital facets there is an anterior invagination. As this area is close to the groove on the ventral side of the opisthotic for (possibly) cranial nerves VII and/or XI. This invagination is situated more dorsally in *O. icenicus*, subdividing the opisthotic facet rather than positioned between the opisthotic and basioccipital facets [16]. The ventrally directed exoccipital facets and relative size of the foramen magnum contribution are similar to MNHNL BM779. However, the antero-posterior elongation is notably different between the supraoccipitals of the two taxa [5].

#### Quadrate

Only the right quadrate was preserved with PIMUZ A/III 5279. It consists of a large, dorsally projected pterygoid lamella and two posteroventrally directed articular surfaces (Fig. 12A, B). The pterygoid lamella is relatively straight as in *Stenopterygius*, and a distinct occipital lamella, as seen in many ophthalmosaurs, is absent [16, 32, 53, 61]. The pterygoid lamella ends ventrally in a small but apparent angular process. This process is occasionally seen in parvipelvians such as *Nannopterygius*, *U. nessovi* and *Arthropterygius volgensis* but is absent in many others including *Stenopterygius*, *O. icenicus* and UAMES 3411 [14, 16, 43, 53, 61, 62]. The stapedial facet is situated closer to the ventral part of the quadrate than to the dorsal edge, as seen in many parvipelvians but notably contrary to the state in UAMES 3411 [14].

#### Articular

The left articular of PIMUZ A/III 5279 is preserved (Fig. 12C). It is quadrangular in outline in medial and lateral view as in non-ophthalmosaurian parvipelvians and many ophthalmosaurs (e.g. *Stenopterygius*, *Ichthyosaurus* and *Mollesaurus*: [15, 42, 50]), but differing from *U. nessovi*, in which the articular is shaped like a parallelogram [43] (Fig. 12C). On the medial surface, the facet for the prearticular is faintly visible posteroventrally. The anterior margin of the articular forms the glenoid contribution. In the reconstruction, the articular partially rests on a ventrolateral facet on the ventromedial surangular, thereby being held in place laterally by the latter element and ventromedially by the prearticular.

#### Epipterygoid

We tentatively identify a left epipterygoid footplate in PIMUZ A/III 5279 (Fig. 7B). The footplate is round in outline in ventral view and the start of the distinct posterodorsal curvature seen in *Stenopterygius* and *Hauffiopteryx* is already apparent [42, 54]. Ossified epipterygoids are present in the basal ichthyopterygian *Chaohusaurus* and the merriamosaurs *Besanosaurus*, *Ichthyosaurus*, *Hauffiopteryx* and *Stenopterygius* [17, 42, 50, 54, 71], but to date have not been reported in any ophthalmosaurian. Whether this represents an evolutionary loss in ophthalmosaurs is unclear as the epipterygoid is often overlooked.

#### Hyoid apparatus

No elements of the hyoid apparatus were recovered.

### Appendicular skeleton

#### Clavicle and interclavicle

**Fig. 13:**
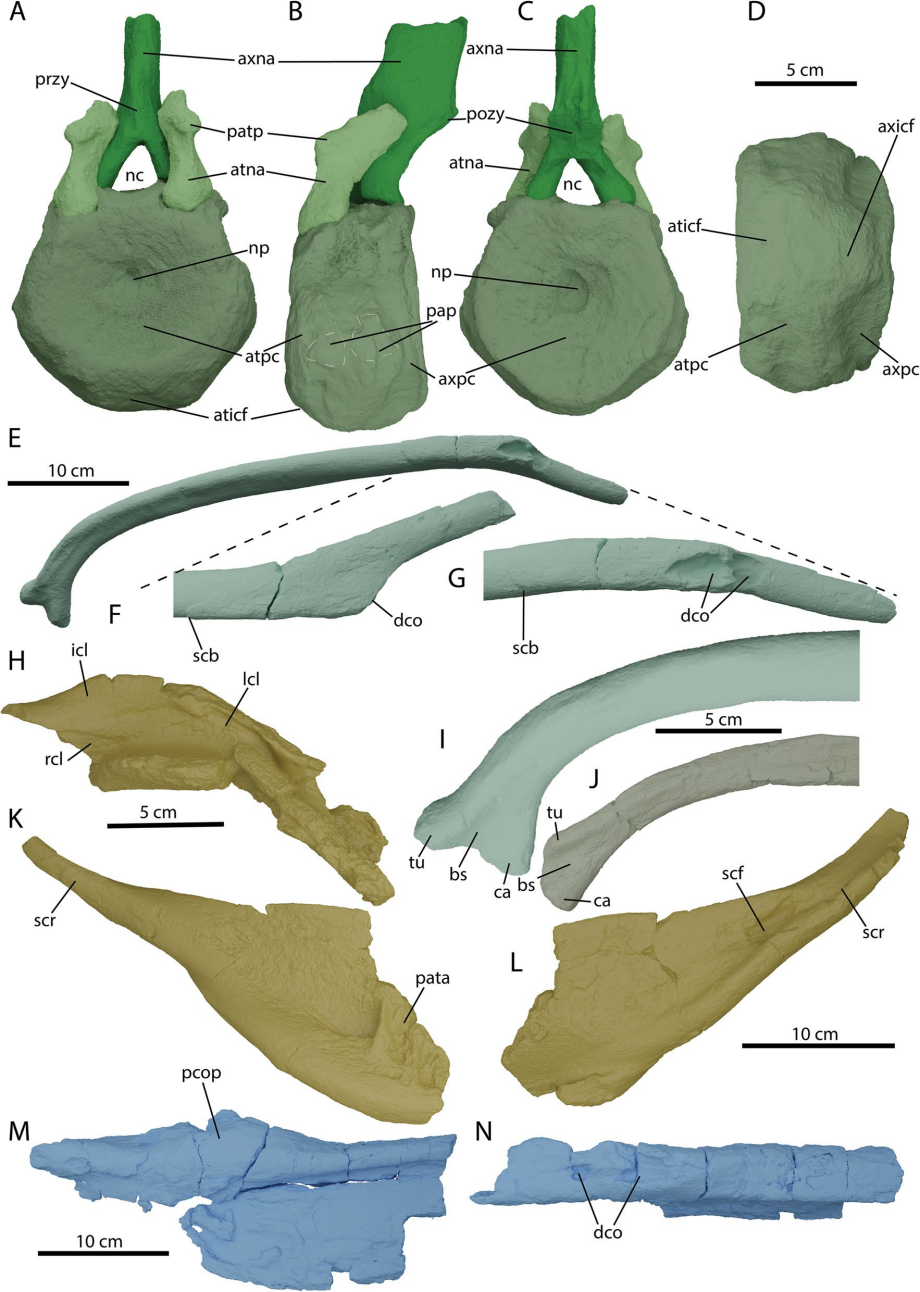
Selected details of the postcranium and the pathological areas of *Argovisaurus martafernandezi*, holotype PIMUZ A/III 5279. Atlas-axis complex in: **A** anterior, **B** left lateral, **C** posterior and **D** ventral view. Complete right dorsal rib with distal pathology: **E** complete in posterolateral view, **F** detail of distal portion of anterior view, **G** detail of distal portion of lateral view. **H** Interclavicle and partial left and right clavicle in medial view. **I** Proximal portion of right anterior dorsal rib in posterior view. **J** Proximal portion of right posterior dorsal rib in posterior view. Right clavicle in: **K** ventral and **L** dorsal view. Pathological area of left surangular: **M** medial and **N** laterodorsal view. Abbreviations: atna, atlas neural arch; aticf, atlas intercentrum facet; atpc, atlas pleurocentrum; axicf, axis intercentrum facet; axna, axis neural arch; axpc, axis pleurocentrum; bs, bony sheet; ca, capitulum; dco, dorsal concavities; icl, interclavicle; lcl, left clavicle; nc, neural canal; np, notochord pit; pap, parapophysis; pata, pathological area; patp, proatlas process; pcop, paracoronoid process; pozy, postzygapophysis; przy, prezygapophysis; rcl, right clavicle; scb, shifted cortical bone; scf, scapular facet; scr, scapular ramus; tu, tuberculum

Both clavicles of PIMUZ A/III 5279 are observed, but the right one is far better preserved than the left (Fig. 13H, K-L). The clavicle consists of a broad medial sheet and a short distally projected rod. This morphology is also seen in *Arthropterygius, Nannopterygius,* and *Thalassodraco* [51, 53, 62]. Although the medial margin of the right clavicle is broken, it was found in contact with the interclavicle and antimeric clavicle and we therefore think the element would not be much longer medially. On the posterior side a distinct depression is visible dorsolaterally, proximal to the distal rod. This depression is the facet for the scapula. Ventrally the clavicle has a distinct boss directed posteriorly holding a short facet, presumably for the interclavicle.

We tentatively identify a partial interclavicle (Fig. 13H). It is fused to the clavicles and preserved on the partial and broken left clavicle, and represents the transverse bar. It is rhomboidal in outline and divided along the midline in external view. A similar morphology was described for *Pl. hercynicus* [72].

### Axial skeleton

#### Atlas-axis complex

The recovered atlas-axis complex of PIMUZ A/III 5279 contains the atlas and axis pleurocentra, the axial neural arch, and the left atlantal neural arch (Fig. 13A-D). The atlas and axis pleurocentra are coossified, as in all ophthalmosaurians with the exception of a specimen referred to *Muiscasaurus* [67]. There is a discernible thickening dorsoventrally extending over the lateral surface, which presumably marks the developmental border between the atlas and axis, but this cannot be described as a suture (Fig. 13B). This morphology is similar to some specimens of *O. icenicus*, but is contrary to *Ichthyosaurus* and *Hauffiopteryx* [4, 16, 22]. On the lateral surface, the parapophyses are only vaguely defined. The axis parapophysis is placed slightly higher than the atlantal parapophysis. The diapophyses are placed at the same height at the dorsal-most margin of the centra in lateral view. They are visible as sequential semilunar bulges at a 45° angle to the main body of the centrum and are connected to the neural arch facet. The anterior (atlantal) articular surface is round in outline and very rugosely textured. A small notochordal pit is visible in the center of the articular facet (Fig. 13A). The posterior (axial) articular surface is much flatter and is more trapezoidal in outline. The edges of the articular surface are likewise much better delineated. The notochordal pit is also much more excavated on the axial articular surface (Fig. 13C). The dorsal surface of the fused pleurocentra consists of the floor for the neural canal and the lateral neural arch facets. The floor for the neural canal is relatively smooth in texture and laterally well delineated by raised ridges. These ridges have a sinusoidal shape and form a constriction in the midsection of the dorsal view, probably indicating the transition from atlas to axis. This is contrary to some specimens of *O. icenicus* in which the canal is straight [16]. The atlas and axis neural arch facets are confluent and very rugose in texture. In ventral view, the fused pleurocentra show a smooth and angled surface, often interpreted as a facet for the atlantal intercentrum (e.g., [16]). The facet for the axis intercentrum is less well developed (Fig. 13D). Ossified intercentra are unknown in Ophthalmosauria.

The atlantal neural arch is unfused along the midline and thereby consists of two halves, the left of which is preserved in PIMUZ A/III 5279. It is triradiate in lateral view, due to the dorsally directed short neural spine, the ventrally directed pedicel, and the anteriorly directed proatlas process (Fig. 13A–C). Ventrally it contains a large pedicel ending in the facet for the pleurocentrum. In lateral and medial view, the proatlas process is ventrally separated from the main body of the neural arch by a distinct groove (Fig. 13A, B). Posterodorsally, on the medial surface a round facet is visible, which represents a postzygapophysis. The axis neural arch consists of a broad spine dorsally and two small pedicels which hold the facets for the pleurocentrum ventrally (Fig. 13A-C). Posteriorly, the postzygapophyses are well developed, but the prezygapophyses are virtually absent. In their position is a rugose, flattened area, which functions as the facets for the atlantal neural hemiarches. An unfused atlas neural arch with protruding proatlas process and a large axial neural spine without prezygapophyses is similar to many non-ophthalmosaurian parvipelvians such as *Hauffiopteryx* and *Ichthyosaurus* [4, 22], but differs from *O. icenicus*, in which the pro-atlas process has been greatly reduced, the axial and atlantal neural spines are similar in size to each other but shorter than succeeding arches, and the axial neural arch has well-developed zygapophyses [16]. In *Catutosaurus gasparinae*, the right and left halves of the atlantal neural arch appear to be unfused, but the neural spine is very broad and a pro-atlas process appears absent. However, the neural arch pedicels are proportionately much smaller than those of the axis ([73]; EM pers. observ.). *Thalassodraco etchesi* was described as having two almost equally large neural spines, of with the atlantal neural spine is much broader than the axial spine. Although the atlantal spine partially overlapped the axial spine, prominent axial zygapophyses were present on the latter [51]. PIMUZ A/III 5279 is therefore much more similar to non-ophthalmosaurian parvipelvians than to the morphologies observed in early-diverging ophthalmosaurians.

The atlantal and axial ribs were not recovered in PIMUZ A/III 5279.

#### Postaxis vertebrae

Dorsal and anterior caudal centra are amphicoelous and generally round in outline in articular view (Fig. 1). Apart from the axis, only cervical vertebra three has a distinct ventral keel (Fig. 1). Rib facets migrate antero-posteriorly from a more anterodorsal to a more anteroventral position on the lateral surfaces of the centra. Rib facets and neural arch facets are distinctly delineated in the more anterior presacral vertebrae, but the distinct delineation fades towards posterior (Fig. 1). Anterior neural arches display a high dorsally rounded neural spine and prominently faceted pre- and postzygapohyses at a 45–90-degree angle. The zygapophyses fade out towards the posterior dorsal and anterior caudal vertebrae. There the neural spines are also more anteriorly rounded. Only 37 presacral vertebral centra (including atlas-axis) and 5 potential anterior caudal vertebral centra are preserved, the latter distinguished based on the presence of a synapophysis ventrally on the lateral sides of the centra. The smallest centrum recovered lacks a synapophysis, but is still round in outline and therefore is apical in position.

#### Ribs

Ribs of PIMUZ A/III 5279 generally show a round outline in cross-section. However, some large dorsal ribs show a single-sided midline groove posteriorly. These ribs are rounded proximally and distally. Rib articulation facets are “sheathed-bicapitate”, in which a distinct tuberculum and capitulum can be observed, but the two processes are connected by a concave sheet of bone (Fig. 13I-J). There is an antero-posterior gradient in the development of the articulation processes. Anteriorly, the capitulum and tuberculum are clearly distinct, but become less distinct in more posterior ribs (Fig. 13I-J). *Stenopterygius*, *O. icenicus* and *Mollesaurus* have distinctly bicapitate presacral ribs with widely separated tubercula and capitula [15, 16, 74], Complete ribs are unfortunately rare in PIMUZ A/III 5279; the longest near-complete rib measures 95 cm in length.

#### Dentition

Only a few teeth (2 recognizable and some fragments) are preserved with the specimen and they are all incomplete. No tooth was observed to contain dentine or enamel; only cementum was preserved. The best-preserved tooth is a complete cementum root (Fig. 8P-O). The root is generally round in outline and tapers somewhat posteroventrally. Distally, a round structure is visible projecting from the main body of the root, which is the pulpal cementum [75]. Posteriorly a large, elongated resorption cavity is visible.

### Pathologies

Several cranial and postcranial elements show aberrant morphologies and surface topologies that can be attributed to reparative osteological reactions in response to traumatic injury. The left anterior dentary, when compared to its counterpart on the right, shows enlarged neurovascular foramina of the ventral-most row on its ventrolateral and anterolateral surface surrounded by thickened bony margins (Fig. 8L-M). However, these minor morphological distinctions between both sides may just reflect some slight degree of morphological variation.

Additional asymmetrical morphologies can be identified when comparing the parietals and surangulars. The cerebral impression on the ventral surface of the parietal is subequal, being more strongly defined on the left parietal compared to the right. The supratemporal ramus and facet are more pronounced on the left compared to the right. The postparietal or occipital ridge is more prominently developed in the right parietal compared to the left. Presence of a distinct occipital ridge has been deemed ontogenetically variable in *Stenopterygius*, but not asymmetrical [42]. Asymmetric development of the mandibular adductor muscles in response to the mandibular injury on the left side could be the cause of the asymmetric parietal morphology (muscle insertions sensu: [50]).

**Fig. 14:**
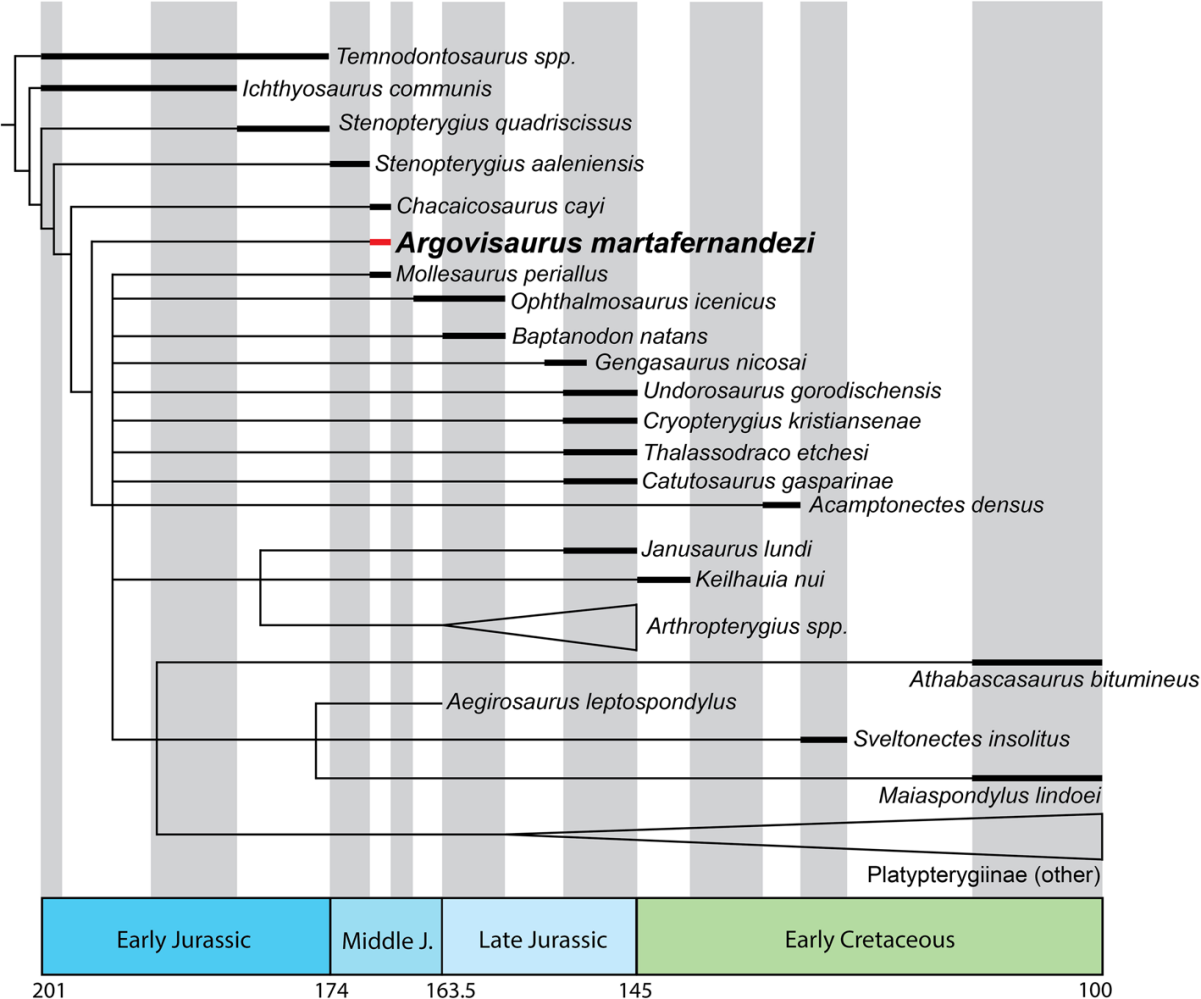
Phylogenetic topology from our second analysis (based on [76]). Thickened lines denote known stratigraphic ranges. Nodes are not placed at known branch points as that wasn’t included in the analysis. The holotype of *Argovisaurus martafernandezi* is resolved between *Chacaicosaurus* and the Ophthalmosauria (*sensu stricto*)

The surangulars are highly divergent in morphology, with the right displaying the normal surface morphology, while the left shows extensive broadening and an overall highly warped paracoronoid area (Figs. 8D, E and 14M, N). Two distinct depressions can be discerned on the dorsolateral margin of the paracoronoid region, a small circular depression (roughly 2 cm along the longest axis and 8 mm deep) and an elongate roughly elliptical depression positioned more anteriorly 55 mm in anteroposterior extent and 5 mm deep (Fig. 8M-N). Overall, the dorsal shelf of the surangular in the paracoronoid area is dramatically mediolaterally expanded resulting in a broadened shelf that slopes anteromedially. The overall surface is characterized by normal cortical textures but aberrant topologies.

The right clavicle shows several depressions medial on the external surface bordered by anteroposteriorly oriented ridges (Fig. 8K).

Additional abnormalities can be observed in the distal part of an anterior dorsal rib along a quarter of its lateral surface. The rib is visibly broadened in this region, attaining a mediolateral width that exceeds that of the proximal portion, being roughly twice that of the adjacent distalmost section. Two large ovoid depressions, separated by an anteroposterior wall, penetrate the surface to a depth of roughly 5 mm at its most extensive (Fig. 13E-G).

### Phylogenetic analysis and implications

In order to assess the phylogenetic position of PIMUZ A/III 5279 within Ichthyosauria, we scored the specimen in the matrix of [77], version modified by [22]. Character ordering settings were as in the aforementioned studies. We ran a tree search in the software package TnT v. 1.5 [78] with maximum parsimony as the optimization criterion. We used a new technology search using the sectorial search, ratchet (50 iterations), and tree fusing search tools and recovered the shortest tree 500 times. This was followed by a traditional search with trees from RAM.

This resulted in > 10 000 trees of length 1672. The strict consensus placed PIMUZ A/III 5279 in a polytomy with all parvipelvians. In order to better analyze the results obtained, we ran the Iter PCR script [79] on the resulting strict consensus tree. This resulted in 55 unstable taxa being pruned from the analysis. PIMUZ A/III 5279 was positioned at the base of Ophthalmosauria.

PIMUZ A/III 5279 grouped in a basal position within Ophthalmosauria, but unambiguously within the clade. The position is supported by two unambiguous synapomorphies in the optimized topology, absence of a sagittal eminence (Character 66) and exclusion of the maxilla from the narial opening (Character 15). We are confident of the ophthalmosaurian affinities of the genus due to the morphology of the angular and basioccipital. We reanalyzed PIMUZ A/III 5279 in the ophthalmosaurian-specific matrix of [76], in order to better resolve its position within Ophthalmosauria. We added *Nannopterygius enthekiodon*, *Catutosaurus gasparinae*, and *Thalassodraco etchesi* to the matrix, since based on the preliminary analysis PIMUZ A/III 5279 is recovered near the base of Ophthalmosauria within ophthalmosaurine-grade ophthalmosaurians, thus these genera are pertinent to better resolving its phylogenetic relationships. Moreover, we rescored characters for the braincase of *Baptanodon natans* based on new information [65]. We ran a tree search in the software package TnT v. 1.5 [78] with maximum parsimony as the optimization criterion. We used a new technology search using the sectorial search, ratchet (50 iterations), and tree fusing search tools and recovered the shortest tree 100 times. This was followed by a traditional search with trees from RAM.

This resulted in 1000 trees of length 249. The strict consensus placed PIMUZ A/III 5279 in a polytomy with all ophthalmosaurians. In order to better analyze the results obtained, we ran the Iter PCR script [79] on the resulting strict consensus tree. *Leninia* was identified as being particularly unstable. Pruning this taxon from the strict consensus resulted in PIMUZ A/III 5279 being resolved between *Chacaicosaurus* and Ophthalmosauria.

This position makes *Argovisaurus* strictly speaking not an ophthalmosaurian following the phylogenetic definition of the clade [31]. However, as this genus matches all morphological characters defining the clade that are sufficiently preserved to be observed, we consider *Argovisaurus* to be an early-diverging ophthalmosaurian.

## Discussion

### Size and ontogenetic stage

Based on its large body size, the smooth basioccipital condyle [61], and well-ossified vertebrae with defined vertebral apophyses [80], PIMUZ A/III 5279 is likely to be at least a large juvenile. The many signs of advanced pathological bone remodeling and perhaps some degree of cranial warping as a result, suggest that the individual survived for many years. It is therefore likely to represent a more advanced ontogenetic stage, and likely was sexually mature. Accompanying histological data would be required to assess full skeletal maturity [81, 82], although extensive endosteal mineral deposition may complicate such studies [83]. Given the late ontogenetic stage of the holotype specimen, we deem the morphological details to be (close to) the true adult morphology, which is useful in light of the phylogenetic analysis.

*Argovisaurus* is quite large, as most baracromian ichthyosaurs do not exceed 1 m in mandibular length or are estimated larger than 4 m in total length. Given the disarticulated nature of the specimen it is difficult to estimate body length. With an approximate mandible length of 1250 mm, an orbital length around 250 mm and a basioccipital width of 121 mm, we estimate a total length somewhere between 4.5–6 m, based on the published data of other baracromian ichthyosaurs (Supplemental Table 1). *Cryopterygius kristiansenae*, *Platypterygius australis, Pl. americanus* and *Brachypterygius*/ *Grendelius* (based on the *Grendelius mordax* holotype) are most similar in cranial length and *Cr. kristiansenae* and *Pl. australis* also have similar orbital sizes [46, 49, 84, 85]. The holotype of *Cr. kristiansenae* is estimated to have attained 5.5 m length, and large specimens of *Pl. australis* are estimated at 6 m in length [46, 85]. Based on more complete material, usually only members of the Platypterygiinae (or Brachypterygiidae, see [31, 33] for discussion) reached those lengths (e.g. *Brachypterygius, Platypterygius* spp*. Parrasaurus;* note: the affinities of *Undorosaurus* spp. are still debated) [46, 72, 85, 86].

Even though mostly fragmentary material exists, some Middle Jurassic ichthyosaurs also seem to have been relatively large. The holotype of *Mollesaurus* has lost its rostral area, but the basioccipital width is slightly wider than that of *Argovisaurus* ([11]; supplemental file 2). *Mollesaurus* is supposed to have an extremely large orbit so true cranial and postcranial lengths are difficult to estimate [11]; however, based on the preserved presacral vertebral column (estimated at 2.5 m based on [11]), a length of up to or exceeding 6 m is possible. *Chacaicosaurus* (= *Stenopterygius*?) also has a large skull (990 mm) [12]. However, its orbital length and basioccipital width are smaller, and the large skull length is largely due to longirostry. UAMES 3411 has a basioccipital width smaller than that of *Chacaicosaurus* (57 mm, remeasured from cast; [14]), and more similar to e.g., *Nannopterygius* (body length estimated at only 3.5 m). *Stenopterygius aaleniensis* is also rather small and thus more similar in size to other members of its genus (ca. 3 m total length) [13]. *Ophthalmosaurus icenicus* is smaller, at only 4 m estimated length [16]. *Argovisaurus* is therefore large compared to other Middle Jurassic taxa from Europe, and comparable in size to *Mollesaurus* from the Eastern Pacific.

### Comparison with other Middle Jurassic ichthyosaurs

Apart from size, there are other differences between *Argovisaurus* and known Middle Jurassic ichthyosaurs. *Argovisaurus* differs from *Mollesaurus* in the morphology of the basioccipital (no raised foramen magnum floor in *Argovisaurus*), stapes (more slender distal process and separated raised opisthotic facet in *Argovisaurus*) and ribs (sheathed-bicapitate ribheads in *Argovisaurus*) [11, 15]. It also differs from *Chacaicosaurus* in relative rostrum length, atlas-axis pleurocentrum morphology (no distinct keel or triangular outline in *Argovisaurus*), and basioccipital morphology (basioccipital anteroposteriorly longer in *Chacaicosaurus*, with a more prominently developed ventral eca) [12]. The specimen referred to as UAMES 3411 is different in basioccipital (longer and with a larger eca than in *Argovisaurus*), stapes (more slender distal process and separated raised opisthotic facet in *Argovisaurus*), and quadrate morphology (slightly more ventrally placed stapedial facet and more pronounced angular process in *Argovisaurus*) [14]. The specimen from the Bajocian of Luxembourg referred to as MNHNL BM779 shares limited anatomical overlap, but differs from *Argovisaurus* in having a proportionately smaller supraoccipital contribution to the foramen magnum, and a much broader dorsal arch [5].

### Ecology and implications for the evolution of the Ophthalmosauria

**Fig. 15:**
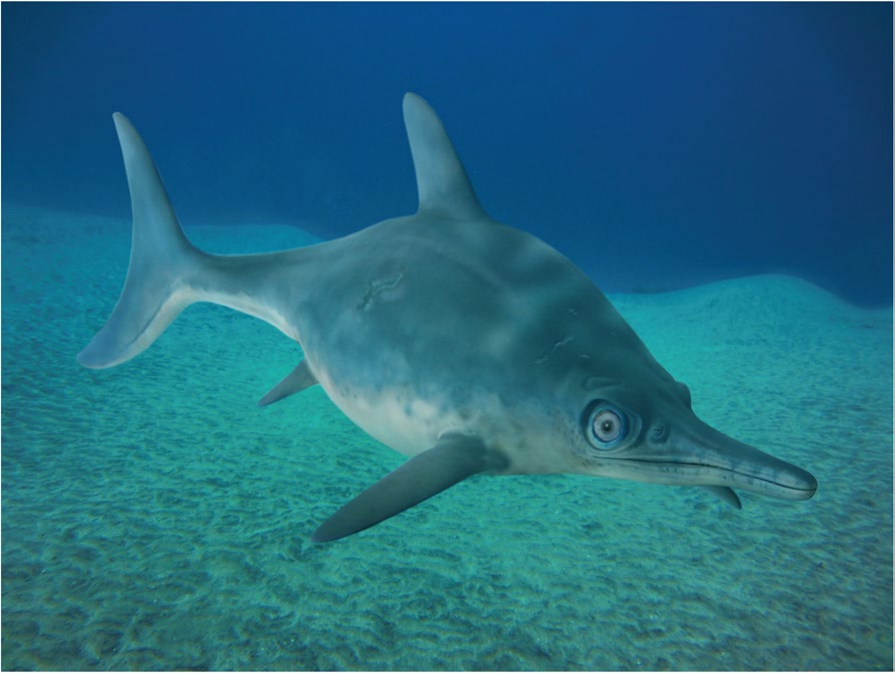
In life reconstruction of *Argovisaurus martafernandezi*. Credit: Beat Scheffold, Winterthur CH

The major ecologically relevant differences between *Argovisaurus* and contemporary taxa are the overall body size, relative size and robusticity of the rostrum as well as the presence of pseudoalveoli in the anterior half of the rostrum and dentary. The combination of these factors suggests *Argovisaurus* was a predator of nektonic macro-invertebrates or vertebrates (Fig. 15). The Middle Jurassic had many large bodied cephalopods including *Megateuthis* and *Lytoceras*, which were found in close proximity with the holotype of *Argovisaurus*. Secondly, as the Middle Jurassic is characterized by a regressive marine event, fewer coastal environments were present and the need for hunting in open water environments was increased [9]. Ophthalmosauria seem to have thus acquired traits for hunting large nekton during a time of limited coastal niches by first evolving large body size and robust snouts for grasping larger prey. Interestingly, this evolutionary trend towards larger body size and the adaptations for hunting larger nektonic prey are mirrored in the Bajocian within Plesiosauria. Large-bodied, macropredatory thalassophonean pliosaurs likewise appear in the early Middle Jurassic [87, 88]. While the morphological traits acquired by *Mollesaurus* and *Argovisaurus* contrast the more closely related, slender-snouted, larger-eyed and generally smaller bodied common hunters of invertebrate nekton in the Early Jurassic (e.g., *Stenopterygius, Hauffiopteryx*), they are consistent with ecomorphology of the more distantly related large Early Jurassic predators such as *Temnodontosaurus* and *Suevoleviathan* and can be viewed as convergent. Many members of the Ophthalmosauria (e.g. *Baptanodon, Aegirosaurus, Nannopterygius* and *Thalassodraco* [41, 51, 53, 89] re-evolved the slender-snouted smaller-bodied morphology in the Late Jurassic as the number of shelf-habitats increased. In the Cretaceous, members of the Platypterygiinae again developed large body sizes and in some cases heterodont dentition, again convergence on pelagic hunting of large nekton [33].

## Conclusion

*Argovisaurus martafernandezi* is a new genus and species of large ichthyosaur from the Bajocian of Switzerland. The holotype and only known specimen (PIMUZ A/III 5279) consists of a nearly complete cranium, fragmentary pectoral girdle, and a large portion of the vertebral column and ribs. This taxon is characterized by a robust snout and anterior pseudoalveoli. We were able, using CT- and surface scanning to make a complete in vivo reconstruction of the cranium. *Argovisaurus* is an important taxon as it sits at the base of the derived ichthyosaur clade Ophthalmosauria. Moreover, Middle Jurassic ichthyosaurs in general are rare as vertebrate-bearing strata are limited. We recognize that the acquisition of a large body size is present at the base of the Ophthalmosauria. We hypothesize that this increase in body size may have allowed early ophthalmosaurians in hunting macronekton at depth. These adaptations mirror the acquisition of macropredatory traits in plesiosaurs as the thalassophonean pliosaurs appear at roughly the same time.

### Supplementary Information

**Additional file 1: Table 1.** Measurements as related to size for adult baracromian ichthyosaurs. Measurements are in mm unless specified otherwise.

## Data Availability

Original material of PIMUZ A/III 5279 is available for study at the PIMUZ, contact curator Christian Klug or Torsten Scheyer for study. Surface meshes of individual elements are available at Morphosource (www.morphosource.org) under the project: Surface scans holotype *Argovisaurus martafernandezi* PIMUZ A/III 5279. Raw CT-scan data are still under study but can be acquired upon direct request from the authors.
